# Differential Inhibition of Human and Trypanosome Ubiquitin E1S by TAK-243 Offers Possibilities for Parasite Selective Inhibitors

**DOI:** 10.1038/s41598-019-52618-3

**Published:** 2019-11-07

**Authors:** D. Roeland Boer, Marie-José Bijlmakers

**Affiliations:** XALOC beamline, ALBA synchrotron (CELLS), 08290 Cerdanyola del Valles, Spain

**Keywords:** Ubiquitylation, Target identification

## Abstract

Novel strategies to target *Trypanosoma brucei, Trypanosoma cruzi* and *Leishmania* are urgently needed to generate better and safer drugs against Human African Trypanosomiasis, Chagas disease and Leishmaniasis, respectively. Here, we investigated the feasibility of selectively targeting in trypanosomatids the ubiquitin E1 activating enzyme (UBA1), an essential eukaryotic protein required for protein ubiquitination. Trypanosomatids contain two UBA1 genes in contrast to mammals and yeast that only have one, and using *T. brucei* as a model system, we show that both are active *in vitro*. Surprisingly, neither protein is inhibited by TAK-243, a potent inhibitor of human UBA1. This resistance stems from differences with the human protein at key amino acids, which includes a residue termed the gatekeeper because its mutation in E1s leads to resistance to TAK-243 and related compounds. Importantly, our results predict that trypanosomatid selective UBA1 inhibition is feasible and suggest ways to design novel compounds to achieve this.

## Introduction

The burden of disease caused by the trypanosomatid parasites *T. brucei*, *T. cruzi* and *Leishmania* is immense, leading to severe disabilities and tens of thousands of deaths every year, predominantly in the poorest countries^1^. Collectively, around half a billion people are at risk of these trypanosomatid infections^1^. The control of infections is extremely difficult because of their transmission by insects and the existence of animal reservoirs for most of these parasites, and effective drug treatments are therefore of the utmost importance. The number of current drugs is very limited and existing treatments have substantial shortcomings in delivery method, efficacy and safety^2^. Therefore, continued efforts to identify and validate novel drug targets and to discover new drugs are ongoing^2^.

Here, we have focused on a potential drug target that is expressed in all eukaryotes, the ubiquitin-activating E1 enzyme (UBA1), with the aim of determining if selective targeting of the trypanosomatid, but not the human protein, is possible. Provided sufficient species selectivity can be found, UBA1 is an attractive drug target because it is essential for cell viability^3–6^ and has two enzymatic activities, ubiquitin-adenylation and ubiquitin-thioester bond formation^6,7^, which can both be inhibited. The function of UBA1 is to activate ubiquitin, which is the first step in ubiquitination, a post-translational modification that attaches one or more ubiquitins to target proteins^8,9^. Proteins that are modified in this way will either be degraded by the proteasome or undergo changes in localization and/or activity^9^. Ubiquitination is a widespread modification that is involved in the regulation of many cellular processes^10^.

**Figure 1:**
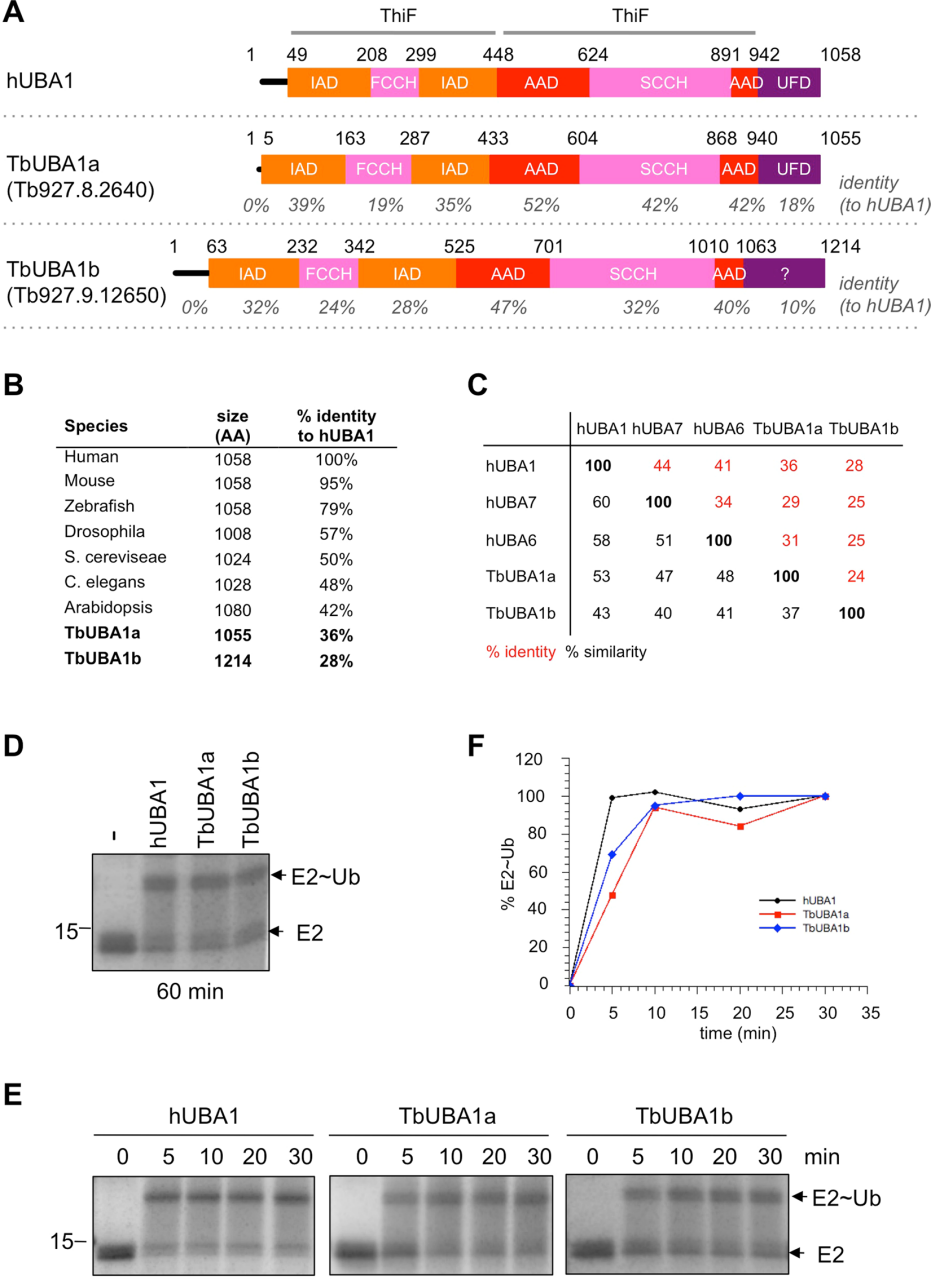
*T. brucei* expresses two UBA1 proteins, TbUBA1a and TbUBA1b. (**A**) Schematic representation of UBA1 proteins showing the domain organization, with domain boundaries indicated above. ThiF refers to the ThiF/MoeB Pfam motif PF00899 by which E1 proteins can be identified. The amino acid identity with hUBA1 was calculated for each domain separately based on pair-wise alignments (listed in grey). (**B**) For the UBA1 orthologues of the indicated species, protein length is indicated as well as the amino acid identity to hUBA1 based on pair-wise alignments. UniprotKB accession numbers: P22314 (human); Q02053 (mouse); F1RCA1 (zebrafish); O46111 (Drosophila); P22515 (*S. cerevisiae*); Q3S1J5 (*C. elegans*); P93028 (Arabidposis); Q57XC5 (TbUBA1a); Q38DE8 (TbUBA1b). (**C**) percentage amino acid sequence identity (in red) and similarity (in black) for the indicated proteins based on pair-wise alignments. (**D**) E1-E2 transthioesterification reactions in the absence (−) or presence of equivalent amounts of the indicated UBA1 proteins. Reactions were run for 60 min. E1 activity was determined by the transfer of ubiquitin to the E2 (UbcH5a). Indicated are free E2 (E2) and the E2-ubiquitin conjugate (E2~Ub). Shown are Coomassie stained gels, with the position of the 15 kD Mr marker band indicated on the left. (**E**) E1-E2 transthioesterification reactions with the indicated UBA1s. Samples were taken before the start of the reaction (0) and after the start of the reaction at the indicated times. Analysis as in (**D**). Reactions were run on separate gels. For full length gels, see Supplementary Information. (**F**) Quantification of the experiment shown in (**E**). For each protein, the E2~Ub as a percentage of the total E2 [E2~Ub/(E2 + E2~Ub)] was calculated. The percentage at 30 min was set at 100%, and the percentages at other time points were calculated relative to this.

UBA1 proteins are multidomain monomers characterized by the presence of two ThiF/MoeB homology motifs, the common building block of E1 proteins that shares sequence homology with the prokaryotic proteins ThiF and MoeB^11^, and a C-terminal Ubiquitin Fold Domain (UFD)^6^ (Fig. 1A). The N-terminal ThiF/MoeB motif contains the inactive adenylation domain (IAD) with primarily a structural role, while the C-terminal ThiF/MoeB motif contains the active adenylation domain (AAD). Inserted inside these ThiF/MoeB domains are the first and second half of the active cysteine domain, the FCCH and SCCH respectively, with the latter containing the catalytic cysteine^12,13^. The activation of ubiquitin requires the covalent attachment of AMP to the C-terminus of ubiquitin, which occurs in the adenylation domain and requires the hydrolysis of ATP^7,14^. Next, the AMP~ubiquitin adduct is attacked by the catalytic cysteine, which leads to a high energy UBA1~ubiquitin thioester conjugate. Following the binding and adenylation of a second ubiquitin, the thioester-bound ubiquitin can be transferred to a ubiquitin conjugating enzyme (or E2) that is recruited via the UFD^13^. A subsequent interaction of the E2 with a ubiquitin ligase (E3), mediates the transfer of ubiquitin to target proteins recruited by the E3^8–10^. Parallel pathways, each with their own designated E1, E2 and E3 proteins, exist for the ubiquitin-like proteins such as SUMO, Nedd8, FAT10, ISG15 and ATG8^6,8^.

The success of the proteasome inhibitor Bortezomib (Velcade®) in the treatment of multiple myeloma^15^ has prompted interest in the discovery of UBA1 inhibitors^16^. The first cell permeable inhibitor was PYR-41 that inhibits the UBA1~ubiquitin thioester bond formation by an unknown mechanism^16,17^. A more recent and considerably more potent inhibitor is TAK-243 (previously called MLN7243) that inhibits human and yeast UBA1 at nanomolar concentrations and has much weaker activity against the E1 proteins of SUMO, Nedd8, ISG15 and ATG8^18^. This adenosyl sulfamate inhibits UBA1 by binding to the ATP-binding site from where it attacks the thioester-bound ubiquitin so that a ubiquitin~TAK-243 adduct is formed that cannot be released, which blocks further ubiquitin activation^18,19^. This mechanism was first elucidated for the related molecule MLN4924^20^, a highly selective inhibitor of the Nedd8 E1 UBA3 that is being tested in phase II clinical trials for specific cancers.

Like in other eukaryotes, the ubiquitin/proteasome system (UPS) of trypanosomatids is essential for viability^21–23^. It is involved in basic cellular processes as expected, but also in trypanosomatid specific processes such as the remodelling of the flagellum during *T. cruzi* differentiation^24^ and tolerance to ionizing radiation^25^. Proteasome inhibition has been shown to kill trypanosomes in animal infection models^26^, providing important *in vivo* support for the therapeutic benefit of targeting the UPS. The inhibition of UBA1s would have as an added advantage to also block non-degradative ubiquitination events that are for instance involved in transcription, DNA damage repair and receptor internalization^10,27–30^.

Two UBA1 genes have been identified in the genome of *T. brucei*^27^, whereas only one is present in yeast and mammals^6^. RNAi knockdown of both genes has been shown to lead to severe growth defects^27,31^ (http://tritrypdb.org), but the encoded proteins and their activity have not yet been investigated. Here, we purified the two *T. brucei* UBA1s, and show that both function as ubiquitin E1s *in vitro*. Strikingly, both proteins were largely resistant to inhibition by TAK-243 and we identified the amino acids responsible for this, which were found to be located at a specific pocket of the adenylation domain. These differences with the human UBA1, which are also present in the UBA1s of *T. cruzi* and *Leishmania*, can be exploited to generate inhibitors that selectively inhibit trypanosomatid UBA1s without affecting human UBA1. Ultimately, this may lead to the discovery of compounds with therapeutic properties against the diseases caused by these parasites.

## Results

### *T. brucei* expresses two active UBA1s

The *T. brucei* genes Tb927.8.2640 and Tb927.9.12650 are the only two that encode for proteins with two ThiF/MoeB motifs (PF00899) as in UBA1^6^ (Fig. 1A). The product of Tb927.8.2640 is 36% identical to human UBA1 (hUBA1), has a similar length, and contains all expected UBA1 domains (Fig. 1A–C, Supplementary Figs S1 and S2). The Tb927.9.12650 gene product is only 28% identical to hUBA1, is longer than most UBA1s, and does not have an annotated UFD in domain databases (Fig. 1A–C, Supplementary Figs S1 and S2). However, Tb927.9.12650 is more closely related to human UBA1 than to human UBA6 and UBA7 (Fig. 1C), the only other E1s with two ThiF domains. Moreover, UBA6, which can also activate ubiquitin, is only expressed in vertrebrates and sea urchin and its other cognate ubiquitin-like (Ubl) protein FAT10 is also not expressed in trypanosomes. Similarly, UBA7 and its Ubl ISG15 with roles in the immune system are not expressed outside of vertrebrates. Further protein BLAST searches revealed that five other E1 orthologues are present in *T. brucei*: the heterodimeric AOS1/UBA2 (SUMO E1) and APPBP1/UBA3 (Nedd8 E1) with UBA2 and UBA3 containing the active sites, and the homodimeric UBA4 (E1 for Urm1 and sulphur transfer), UBA5 (Ufm1 E1) and ATG7 (activates Ubls involved in autophagy) (Supplementary Fig. S3). The sububits of these E1s contain each only one ThiF domain, and their catalytic Cys domains are shorter than that of UBA1. Phylogenetic analysis showed that Tb927.9.12650 is more closely related to Tb927.8.2640 than to these other probable E1s (Supplementary Fig. S3). The overall similarity between Tb927.8.2640 and Tb927.9.12650 is only 24% but this is 48% at their AADs. Thus, based on the above Tb927.9.12650 is most likely to encode for a second UBA1 in *T. brucei*.

To determine ubiquitin E1 activity experimentally, we cloned both Tb927.8.2640 and Tb927.9.12650 genes for expression in *E. coli* and purified the recombinant proteins (Supplementary Fig. S4), which we called TbUBA1a and TbUBA1b, respectively. These were then used in E1-E2 transthioesterification assays (Supplementary Fig. S5), using human ubiquitin (the *T. brucei* homologue is 95% identical, Supplementary Fig. S6) and human E2 UbcH5a (*T. brucei* has a probable orthologue that is 73% identical, Supplementary Fig. S6). Human UBA1 was used as a positive control. The ubiquitin E1 activity is detected in these assays by the transfer of ubiquitin from the E1 to the E2, and this was observed to occur with both TbUBA1a and TbUBA1b (Fig. 1D). Next, time course experiments were performed, which showed that TbUBA1a and TbUBA1b reactions proceeded at comparable rates and were both complete at 10 min (Fig. 1E,F). Therefore, we conclude that the *T. brucei* genome contains two UBA1s with similar *in vitro* activity.

### Structural modelling and *in silico* expression analysis of TbUBA1a and TbUBA1b

**Figure 2:**
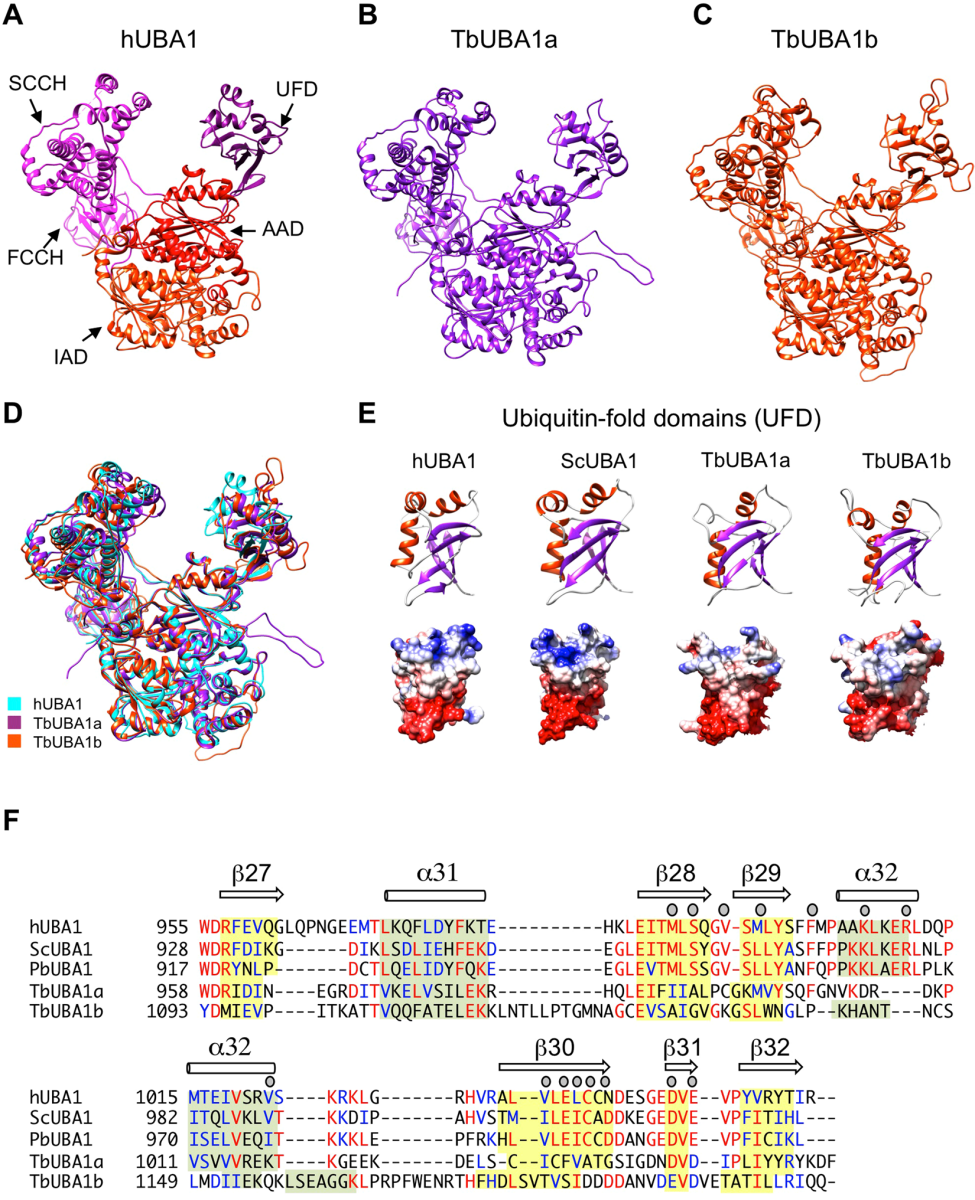
Structural models of TbUBA1a and TBUBA1b predict an UFD in both. (**A**) Ribbon diagram of hUBA1 (PDB 6DC6, chain A) with the separate domains indicated. Molecular graphics and analyses in this figure were performed with UCSF Chimera^42^. (**B**) Structural model of TbUBA1a generated with Phyre2^32^. (**C**) Structural model of TbUBA1b generated with Phyre2^32^. (**D**) Overlay of hUBA1, TbUBA1a and TbUBA1b. (**E**) Comparison of the UFDs of hUBA1, *S. cerevisiae* UBA1 (ScUBA1, PDB 4NNJ), TbUBA1a and TbUBA1b. The side of the UFD that interacts with the E2 is depicted. Top: ribbon diagram with alpha helices (orange) and beta sheets (purple) highlighted. Bottom: surface rendering with coloumbic surface colouring by electrostatic potential. Red: negatively charged; blue: positively charged. (**F**) Amino acid alignment of the UFDs in (**E**) plus the UFD of *Schizosaccharomyces pombe* (PbUBA1, UniProt ID O94609). Identical amino acids are in red, similar amino acids in blue. Similarity groups are (G,A), (I,L,M,V), (D,E), (H,K,R), (W,Y,F) and (S,T). Structural elements (beta sheets and alpha helices) are indicated above the sequence based on structures of the human (PDB 6DC6), *S. cerevisiae* (PDB 4NNJ) and *S. pombe* (PDB 4II3) UBA1s. The beta sheet residues are highlighted in yellow, alpha helix residues in light green. Predicted beta sheets and alpha helices based on the models have been highlighted for TbUBA1a and TbUBA1b as well. Residues that have been shown to interact with E2s for the *S. pombe* UBA1 in PDB 4II2^13^ and 5KNL^34^ are indicated by grey circles.

To further characterize the *T. brucei* UBA1s, and in the absence of having successfully generated protein crystals so far, the protein fold recognition server Phyre2^32^ was used to obtain structural models of TbUBA1a and TbUBA1b. For TbUBA1a, 95% of amino acids were modelled at >90% accuracy (Fig. 2B), for the longer TbUBA1b with an N-terminal extension (Fig. 1A), 85% of amino acids were modelled at the same accuracy (Fig. 2C). The models of these proteins fully overlapped with the structure of the recently crystallized hUBA1^33^ with the exception of loops that represent insertions in TbUBA1a and TbUBA1b (Fig. 2A,D). Moreover, the models provided evidence that like for TbUBA1a, the C-terminal region of TbUBA1b can be folded into an UFD, despite its low sequence identity (10%) with hUBA1 in this region (Fig. 2A–E). In a magnified view these UFDs can be seen to resemble those of hUBA1^33^ and *S. cerevisiae* UBA1^12^ (ScUBA1) demonstrating a typical beta-grasp fold (Fig. 2E). The UFDs of both TbUBA1a and TbUBA1b show very limited sequence similarity to the UFDs of human and yeast UBA1 and also most of the amino acids that make contact with E2s in the *S. pombe* UBA1^13,34^ are not conserved (Fig. 2F). Despite this, the overall charge distributions at the side of the UFD that binds to the E2 are comparable^13,34^ and a negatively charged region that is important for binding to ubiquitin E2s^12^ can be seen close to the C-terminus (Fig. 2E, bottom row). In summary, these models further support that both TbUBA1a and TbUBA1b are orthologues of UBA1 in *T. brucei*.

The expression of TbUBA1a and TbUBA1b has been characterised by RNAseq; both mRNAs are constitutively expressed in the bloodstream form as well as the procyclic (insect) form^35^ (Supplementary Fig. S7, http://tritrypdb.org). Furthermore, a large-scale RNAi silencing study showed that TbUBA1a knockdown resulted in a 55% reduction of *T. brucei* viability within five days, whilst knockdown of TbUBA1b led to a 90% reduction^31^ (Supplementary Fig. S7, http://tritrypdb.org). The silencing of TbUBA1b ranks within the 1 percentile of severest with respect to loss of fitness amongst almost 10,000 genes targeted in this study. Thus, it appears that both *T. brucei* UBA1s are important for *T. brucei* growth but TbUBA1b may play a dominant role.

### TbUBA1s are resistant to inhibition by TAK-243

**Figure 3:**
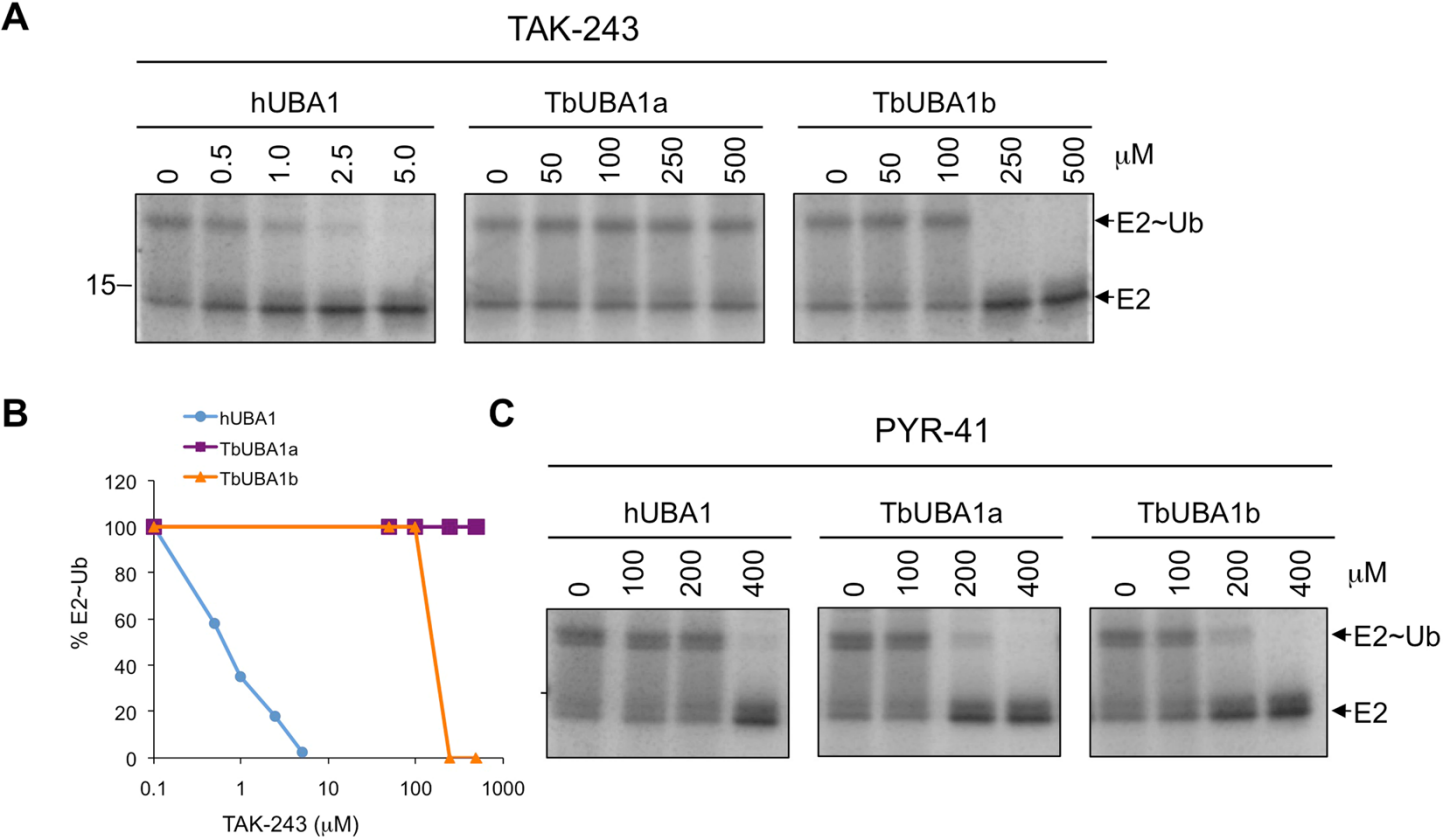
TbUBA1a and TbUBA1b are resistant to TAK-243 inhibition. (**A**) E1-E2 transthioesterification reactions with the indicated UBA1s in the absence (0) or presence of increasing concentrations of TAK-243. Note the difference in concentrations of TAK-243 used for hUBA1 on the one hand, and TbUBA1a and TbUBA1b on the other hand. Reactions were stopped after 30 min. E1 activity was determined by the transfer of ubiquitin to the E2 (UbcH5a). Indicated are free E2 (E2) and the E2-ubiquitin conjugate (E2~Ub). Images are derived from the same Coomassie stained gel. See Supplementary Information for an image of the full length gel. (**B**) Quantification of the experiment shown in (**A**). E2~Ub was calculated as a percentage of the total amount of E2 [E2~Ub/(E2 + E2~Ub)]. This was set at 100% for the reaction in the absence of TAK-243 (0) for each protein separately. (**C**) E1-E2 transthioesterification reactions as in (**A**) performed in the absence (0) or presence of increasing concentrations of PYR-41. Images are derived from the same Coomassie stained gel. See Supplementary Information for an image of the full length gel.

We next aimed to establish whether selective inhibition of the *T.brucei* UBA1s, as opposed to hUBA1, is achievable. To start, we compared the inhibition of the *T. brucei* and human UBA1s by the commercially available selective UBA1 inhibitor TAK-243^18^. TAK-243 is a potent *in vitro* inhibitor of hUBA1^18,19^, and has an anti-proliferative effect against tumour cell lines *in vitro* and against xenograft human tumour cells in mice^18^. This compound acts as an ATP mimetic and binds to the adenylation site of hUBA1^18,19^. To test inhibition by TAK-243, E1-E2 transthioesterification assays were performed following a 30 min pre-incubation with the inhibitor in the absence of Mg~ATP. The reactions were then started by the addition of Mg~ATP and ran for 30 min to completion. As expected, a strong inhibition of hUBA1 by TAK-243 was detected (Fig. 3A,B, IC50 0.8 μM). However, TbUBA1a was not inhibited at the highest concentration tested (500 μM) and is thus at least 500x less sensitive to inhibition by TAK-243 than hUBA1 (Fig. 3A,B). TbUBA1b was also resistant to TAK-243, although not to the same extent as TbUBA1a, but required at least 100x higher TAK-243 concentrations than hUBA1 (Fig. 3A,B, IC50 159 μM). Next, we tested the unrelated UBA1 inhibitor PYR-41 that in contrast to TAK-243 does not affect the adenylation of ubiquitin, but inhibits thioester-bond formation of ubiquitin and the E1 instead^17,36^. Therefore, PYR-41 is unlikely to bind to the adenylation site, at least not in the same way as TAK-243, although its precise mode of action has not yet been elucidated. With this inhibitor, that has a lower potency than TAK-243, we observed very little difference between inhibition of human and the T. brucei UBA1s (Fig. 3C). In fact, the TbUBA1s were slightly (~two-fold) more sensitive to PYR-41 than hUBA1. Most importantly, we conclude that there is no general impairment of TbUBA1 inhibition in these assays, and the striking resistance to TAK-243 but not to PYR-41 predicts that important differences between hUBA1 and the TbUBA1s can be found at their ATP-binding sites.

### HUBA1 and TbUBA1s differ at important TAK-243 binding residues

**Figure 4:**
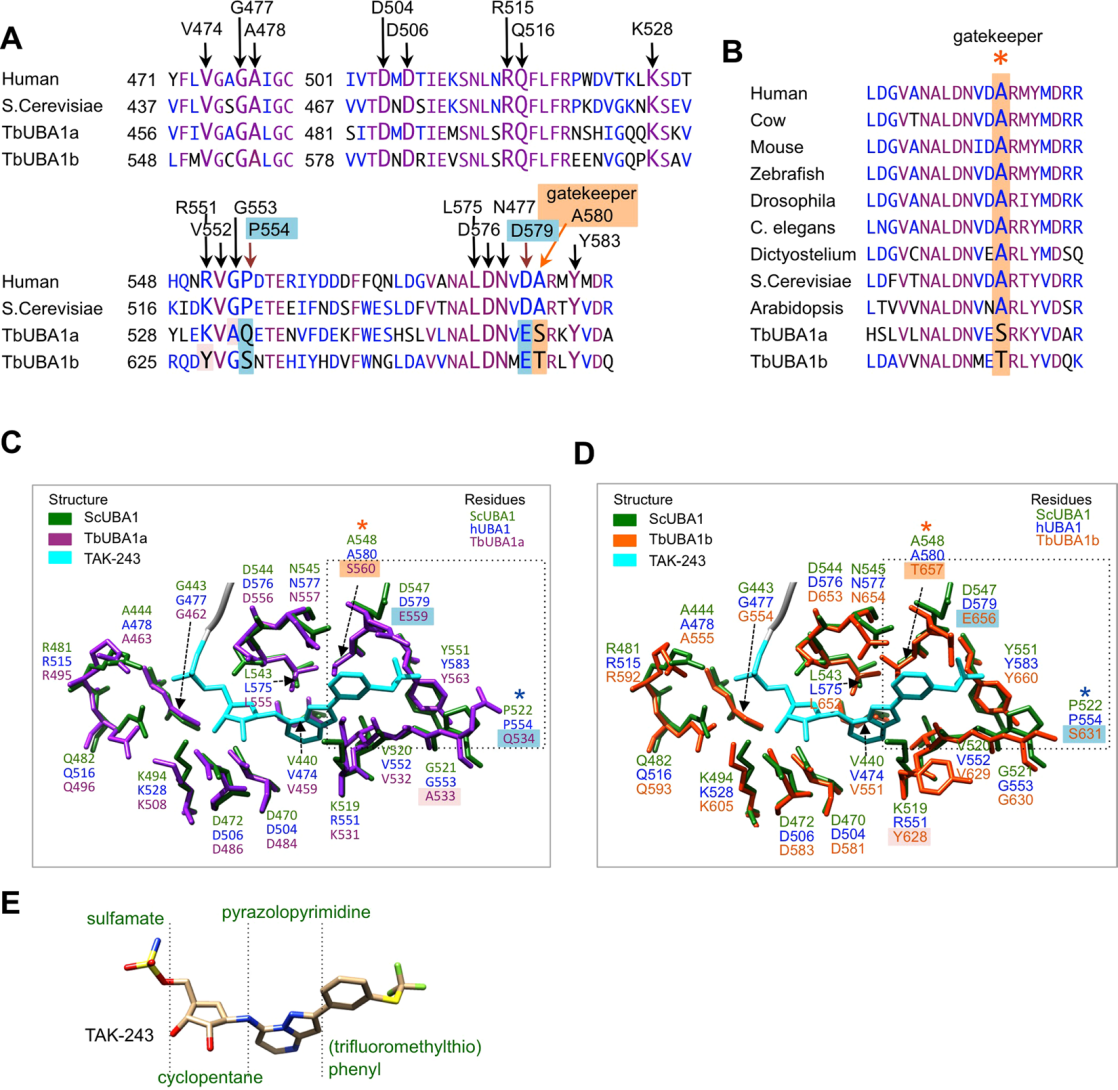
TbUBA1a and TbUBA1b differ from ScUBA1 and hUBA1 at important TAK-243 interacting residues. (**A**) Amino acid alignments showing residues that interact with TAK-243 in larger font and indicated with arrows. Interactions with TAK-243 are based on ScUBA1 in PDBs 5L6J and 5TR4. The labels above the alignment refer to the equivalent amino acids in hUBA1. Residues that are identical in all four sequences are in purple, residues that are at least 50% similar are in blue. Similarity groups are (G,A), (I,L,M,V), (D,E), (H,K,R), (W,Y,F) and (S,T). The *T. brucei* UBA1 residues that were investigated in this study are highlighted with a lightblue background and an orange background for the gatekeeper residue. (**B**) Amino acid sequence alignment of the region surrounding the gatekeeper residue. The alignment shows the high evolutionary conservation in this area and the sequence divergence at the gatekeeper residue for TbUBA1a and TbUBA1b. Colour coding is as in (**A**). (**C**) View of the ScUBA1 adenylation site showing only the ScUBA1 residues that interact with TAK-243~ubiquitin (green), in an overlay with the corresponding residues of TbUBA1a (purple). The numbers of the equivalent residues in hUBA1 are given in blue. The two C-terminal Gly of ubiquitin are shown in grey and TAK-243 is in cyan. Analysis and graphics were made with UCSF Chimera^42^ using PDB 5L6J for ScUBA1 (chain A) bound to ubiquitin-TAK-243 (chain B) and a structural model of TbUBA1b (see Fig. 2B). The labels of TbUBA1a residues that differ from ScUBA1 are highlighted as in (**A**): S560 is at the gatekeeper position and was mutated in this study; Q534 and E559 were also mutated; A533 was not mutated. The area where the (trifluoromethylthio)phenyl group binds is boxed. (**D**) An overlay of TAK-243 residues of ScUBA1 (green) with the corresponding residues of TbUBA1b (orange). Details are the same as in (**C**). Residue labels that are highlighted for TbUBA1b indicate amino acids that differ from ScUBA1. T657 is at the gatekeeper position and was mutated in this study; S631 and E656 were also mutated; Y628 was not mutated. (**E**) structure of TAK-243 (from PDB 5L6J) with carbon (beige), oxygen (red), nitrogen (blue), sulphur (yellow), and fluor (green).

To understand why the *T. brucei* UBA1s are not inhibited by TAK-243, we compared the structural models of TbUBA1a and TbUBA1b with a crystal structure of the *Saccharomyces cerevisiae* UBA1 (ScUBA1) in complex with TAK-243 and ubiquitin^19^. TAK-243 occupies the ATP-binding site of ScUBA1 where it is bound covalently to ubiquitin through the sulfamate nitrogen group (Supplementary Fig. S8). Eighteen amino acids of ScUBA1 interact with TAK-243^19^, and all of these are conserved in hUBA1 with the exception of K519, which is an arginine (R551) in hUBA1 (Fig. 4A–D). A ScUBA1a with a K519R substitution showed similar TAK-243 binding^18^. Consistent with this similarity between hUBA1 and ScUBA1, TAK-243 inhibits hUBA1 and ScUBA1 at comparable concentrations and molecular dynamic simulations showed that the inhibitor binds to hUBA1 and ScUBA1 in similar ways^19^. TAK-243 binds UBA1 at the same location as the AMP of adenylated ubiquitin, but makes additional contacts because of its greater size and protrudes further into the adenylation domain^19^ (Supplementary Fig. S9). The (trifluoromethylthio)phenyl (C_6_H_4_-SCF_3_) group that is most distant from the covalently bound ubiquitin (Fig. 4E) occupies a pocket that is lined by P522, D547, A548 and Y551 in ScUBA1^19^ (Fig. 4C,D). We will refer to these residues by their corresponding positions in hUBA1: P554, D579, A580 and Y583 (Fig. 4A–D). Both TbUBA1s differ at four residues that in ScUBA1 and hUBA1 interact with TAK-243, and strikingly, three of these differences are located in the pocket where the C_6_H_4_-SCF_3_ group binds (Fig. 4A,C,D). Specifically, at the position of P554 in hUBA1, a Gln (Q534) is present in TbUBA1a, and a Ser (S631) in TbUBA1b; at D579 of hUBA1, a Glu is found in both TbUBA1a (E559) and TbUBA1b (E656); most remarkably, at the position of A580 in hUBA1, the so-called gatekeeper residue, a Ser (S560) is present in TbUBA1a and a Thr (T657) in TbUBA1b. The gatekeeper is a highly conserved Ala in UBA1 (Fig. 4B) and its mutation to bulkier amino acids, such as a Thr or Ser, has been shown to impair inhibition of hUBA1 by TAK-243^19,37^. Thus, from these comparisons we can conclude that the two *T. brucei* UBA1s differ considerably from the human UBA1 at residues that are important for TAK-243 binding and that these differences concentrate in one specific pocket and include the gatekeeper residue.

### Crucial residues for the resistance of TbUBA1a and TbUBA1b to TAK-243 inhibition

**Figure 5:**
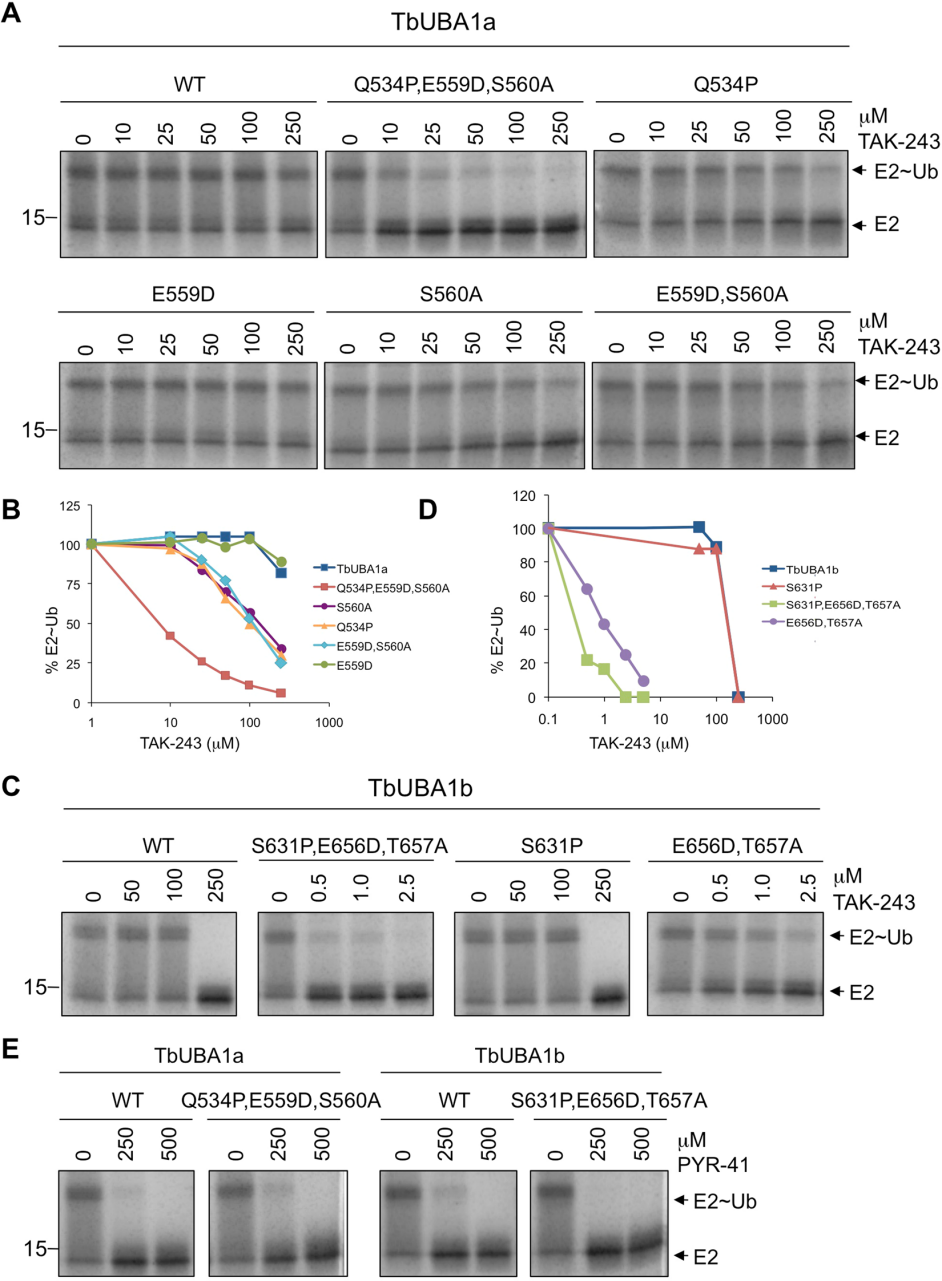
Identification of residues that contribute to TAK-243 resistance of TbUBA1a and TbUBA1b. (**A**) E1-E2 transthioesterification reactions with either wild type TbUBA1a (WT) or TbUBA1a carrying the indicated mutations in the absence (0) or presence of increasing concentrations of TAK-243. Reactions were run for 30 min. E1 activity was determined by the transfer of ubiquitin to the E2 (UbcH5a). Indicated are free E2 (E2) and the E2-ubiquitin conjugate (E2~Ub). Parts of Coomassie stained gels are shown. Images of full length gels are displayed in the Supplementary Information. Reactions with, respectively, WT and the mutant Q534P,E559D,S560A, mutants Q534P and E559D,S560A, and mutants E559D and S560A, were run on the same gel. (**B**) Quantification of the experiment shown in (**A**). E2~Ub was calculated as a percentage of the total amount of E2 [E2~Ub/(E2 + E2~Ub)]. This was set at 100% for the reaction in the absence of TAK-243 (0) for each protein separately. (**C**) E1-E2 transthioesterification reactions with either wild type TbUBA1b (WT) or TbUBA1b mutants. Reactions and analysis as in (**A**). Note the difference in TAK-243 concentration range for WT and S631P on the one hand, and the double (E565D,T657A) and triple (S631P,E565D,T657A) mutants on the other hand. Reactions with, respectively, WT and the mutant S631P, and the mutants S631P,E565D,T657A and E565D,T657A, were run on the same gel. For full length gels see Supplementary Information. (**D**) Quantification of the experiment shown in (**C**). E2~Ub was calculated as a percentage of the total amount of E2 [E2~Ub/(E2 + E2~Ub)]. This was set at 100% for the reaction in the absence of TAK-243 (0) for each protein separately. (**E**) The wild type (WT) and triple mutants of TbUBA1a (left) and TbUBA1b (right) were analyzed in E1-E2 transthioesterification reactions in the absence or presence of PYR-41 as indicated. Reactions and analysis as in (**A**). The TbUBA1a and TbUBA1b proteins were run on separate gels. For full length gels see Supplementary Information.

To investigate the relevance of these amino acid differences between the *T. brucei* and human UBA1s, we generated TbUBA1a and TbUBA1b mutants in which these residues were substituted by their human equivalents. Indeed, the simultaneous introduction of Q534P, E559D and S560A substitutions in TbUBA1a resulted in a large increase in inhibition by TAK-243 (Fig. 5A,B). This triple mutant was inhibited at 10 μM TAK-243 (IC50 12 μM), a more than 25x increase in inhibition compared to wildtype TbUBA1a. When mutations at single residues were introduced, the E559D substitution was found to have no effect, but both the individual Q534P and S560A substitutions resulted in an increase in inhibition by TAK-243 (Fig. 5A,B). These effects were intermediate to that of the triple mutant, suggesting that both Q534 and S560 play important roles in TAK-243 resistance. In the case of TbUBA1b, the simultaneous introduction of S631P, E656D and T657A mutations resulted in a large increase in TAK-243 sensitivity as well (Fig. 5C,D). This triple mutant was inhibited at concentrations at least 200x lower than that required for wildtype TbUBA1b. The increase in inhibition of the double mutant E656D, T657A was almost as large as that of the triple mutant, showing the importance of the T657 at the gatekeeper position (Fig. 5C,D). The E656D mutation was not expected to have an effect given the result with E559D in TbUBA1a. The S631P mutation on its own showed no difference with the wildtype TbUBA1b at the concentration range tested, but contributed to a ~2x increase in TAK-243 inhibition based on the difference observed between the double and triple mutants (Fig. 5C,D). For both TbUBA1a and TbUBA1b, the triple mutations did not affect inhibition by PYR-41, consistent with a selective interference of the mutated residues with TAK-243 binding (Fig. 5E).

**Figure 6:**
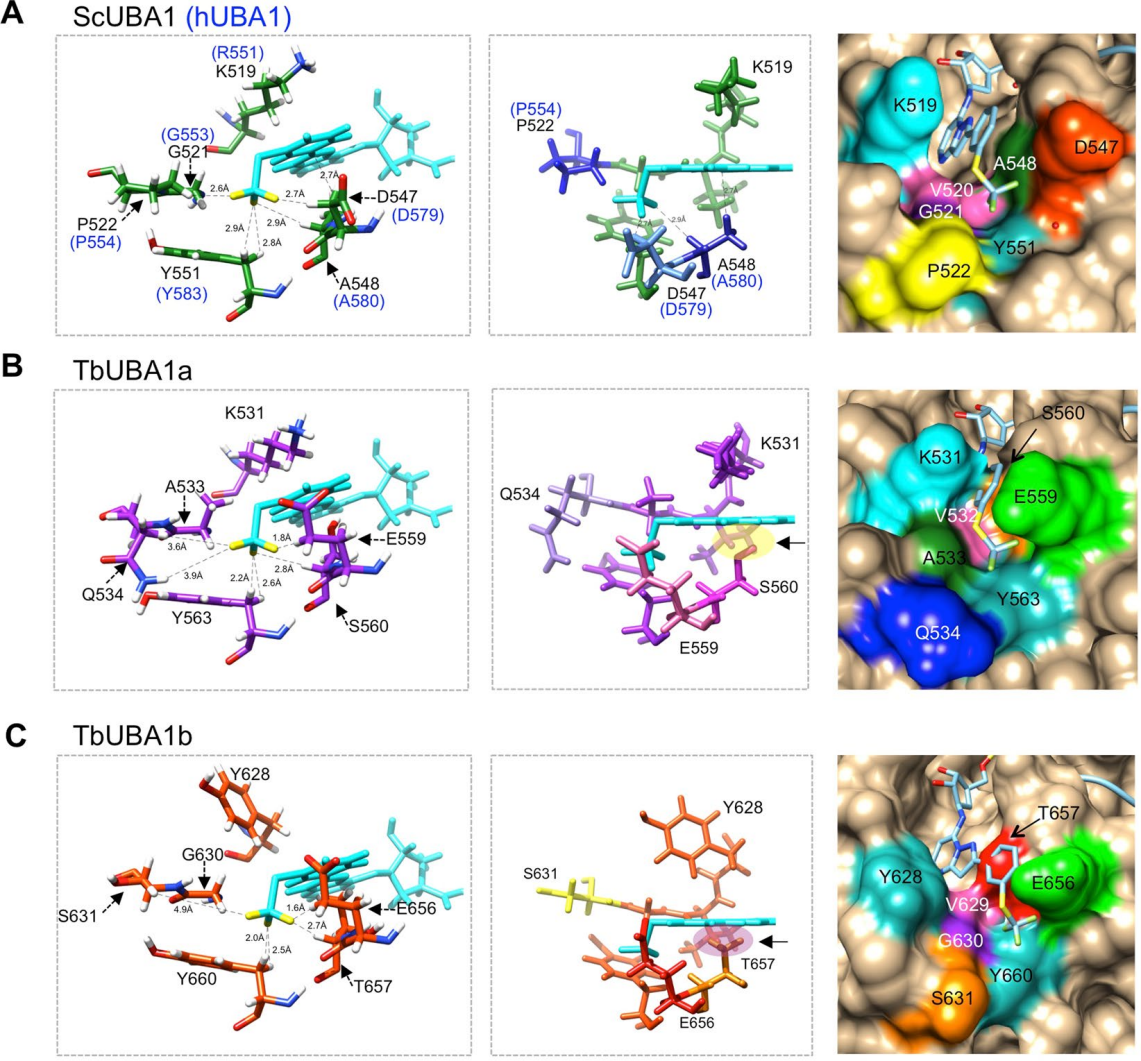
TbUBA1a and TbUBA1b residues interfere with binding to the (trifluoromethylthio)phenyl group of TAK-243. (**A**) Left: magnified view of ScUBA1 showing the interactions between P522, D547, A548 and Y551 with the fluor atoms of TAK-243, based on Misra *et al*., PDB 5L6J. Distances of the interactions are indicated. Human equivalent residues are given in blue; Middle: slightly tilted view to show the distance between the gatekeeper residue A548 of ScUBA1a (A560 in hUBA1) and the phenyl group of TAK-243; Right: space-fill representation of the TAK-243 binding site of ScUBA1. Graphics were generated using UCSF Chimera^42^. (**B**) The same as in A, for the model of TbUBA1a. The distances in the panel on the left were calculated using UCSF Chimera^42^; The panel in the middle shows the clash between S560 of TbUBA1a and the phenyl group of TAK-243 (yellow oval). The panel on the right also shows the clash between TAK-243 and the residues of TbUBA1a due to the short distance between S560 and K531. (**C**) The same as in A, for the model of TbUBA1b. Distances between TbUBA1b residues and TAK-243 were calculated as in B with UCSF Chimera^42^. The panel in the middle shows the clash between T657 of TbUBA1b and the phenyl ring of TAK-243 (purple oval).

To understand the role of these amino acids in more detail, we modelled TAK-243 onto the structural models of TbUBA1a and TbUBA1b, based on overlays with the structure of the ScUBA1/TAK-243~ubiquitin complex^19^. In the case of TbUBA1a, this showed that Q534 is likely to fail to contact the fluorine atoms of SCF_3_, one of which interacts with the equivalent P522 of ScUBA1 (P554 of hUBA1) (Fig. 6A,B, left panels). A similar situation exists for S631 at this position in TbUBA1b (Fig. 6A,C, left panels). Furthermore, the residues at the gatekeeper position, S560 in TbUBA1a and T657 in TbUBA1b, can be seen to clash with the phenyl group of C_6_H_4_-SCF_3_ (Fig. 6A–C, middle panels). A surface rendition of this area also reveals the differences in shapes of this pocket between ScUBA1 and the *T. brucei* UBA1s (Fig. 6A–C, right panels).

To summarize, the presence of Q534 and S560 in TbUBA1a, and primarily T657 in TbUBA1b impair inhibition by TAK-243. This is consistent with the important roles of the equivalent residues in ScUBA1 and hUBA1 for binding the C_6_H_4_-SCF_3_ group of TAK-243^19^. Importantly, this shows that there are substantial structural differences between hUBA1 and TbUBA1s, and that these are concentrated in one specific area of the adenylation domain. Because of this, it is feasible that TAK-243 derivatives can be designed that inhibit the *T. brucei* but not the human UBA1. This is particularly the case for TbUBA1b, the protein with the greatest role in viability. Whereas the space in TbUBA1a appears restricted around the gatekeeper residue, this is not the case for TbUBA1b because of the presence of Y628 (Fig. 6C). Furthermore, the pocket of TbUBA1b lined by G630, S631, Y660, T657 is longer than that of hUBA1 because of the presence of G630 at the position of P554 in human. The absence of a Pro will also lend more flexibility to this pocket and thus may allow the accommodation of a wider range of chemical groups in TbUBA1b than in hUBA1.

### Differences with hUBA1 are also present in the same region of *T. cruzi* and Leishmania UBA1s

**Figure 7:**
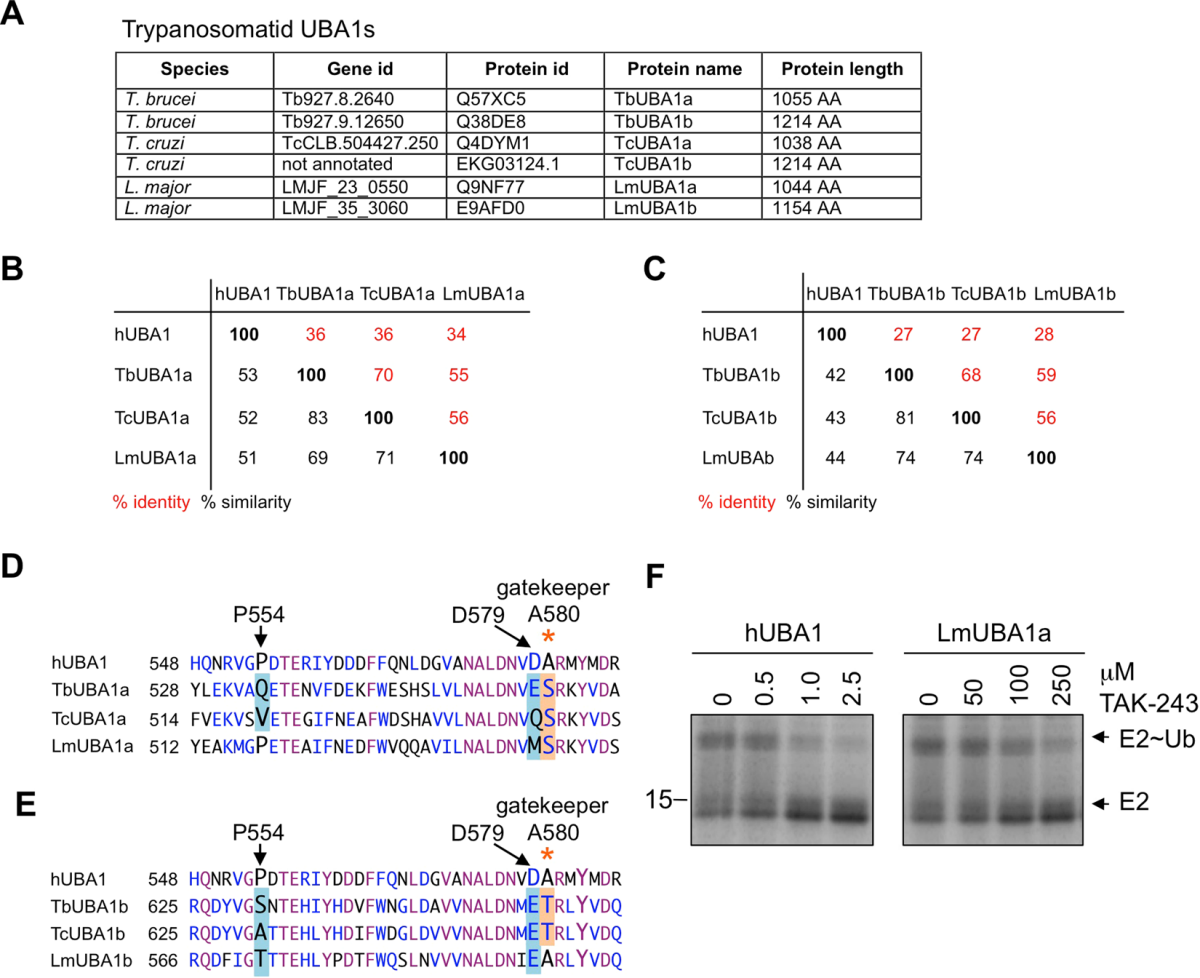
The UBA1 proteins of *T. cruzi* and *L. major* differ from hUBA1 at TAK-243 interacting residues as well. (**A**) Orthologues of TbUBA1a and TbUBA1b identified in *T. cruzi* and *L. major* by BLAST searches. TriTrypdb Gene IDs, UniprotKB accession numbers and protein lengths are given. The proteins were named in this study. (**B**) TbUBA1a orthologues in *T. cruzi* and *L. major*: the percentage of amino acid identity (red) and similarity (black) is based on pair-wise alignments. (**C**) TbUBA1b orthologues in *T. cruzi* and *L. major*: the percentage of amino acid identity (red) and similarity (black) is based on pair-wise alignments. (**D**) Amino acid alignments showing TAK-243-interacting residues of hUBA1 in larger font and indicated by arrows. Differences between hUBA1, TbUBA1a and its orthologues TcUBA1a and LmUBA1a are highlighted. Residues that are identical in all four sequences are in purple, residues that are at least 50% similar are in blue. Similarity groups are (G,A), (I,L,M,V), (D,E), (H,K,R), and (S,T). (**E**) As (**D**), but for TbUBA1b and its orthologues TcUBA1b and LmUBA1b. (**F**) E1-E2 transthioesterification reactions with hUBA1 and LmUBA1a in the absence (0) or presence of increasing concentrations of TAK-243. Reactions were run for 30 min. E1 activity was determined by the transfer of ubiquitin to the E2 (UbcH5a). Indicated are free E2 (E2) and the E2-ubiquitin conjugate (E2~Ub). Images are derived from the same Coomassie stained gel. See Supplementary Information for an image of the full length gel. Note the difference in TAK-243 concentration range for hUBA1 and LmUBA1.

Finally, we investigated whether similar differences are present in the UBA1s of the two other major human disease-causing trypanosomatids, *T. cruzi* and *Leishmania*. We found that these pathogens also contain two UBA1 genes that encode for orthologues of TbUBA1a and TbUBA1b and we named these proteins accordingly (Fig. 7A, Supplementary Fig. S2). The *T. cruzi* UBA1s TcUBA1a and TcUBA1b are ~70% identical to their respective *T. brucei* orthologues, whereas the UBA1s of the more distantly related *Leishmania major*, LmUBA1a and LmUBA1b, are 55% and 59% identical to TbUBA1a and TbUBA1b, respectively (Fig. 7B,C). The two *T. cruzi* proteins differ from hUBA1 at the same three residues in the C_6_H_4_-SCF_3_-binding pocket as the *T. brucei* UBA1s (Fig. 7D,E, Supplementary Fig. S2). Similar to TbUBA1a, a Ser is present at the gatekeeper position of TcUBA1a, and similar to TbUBA1b, a Thr is present at this position in TcUBA1b. The *Leishmania* UBA1s on the other hand, differ at only two of these residues. LmUBA1a differs at the equivalents of human D579 and the gatekeeper A580, but not at P554 (Fig. 7D, Supplementary Fig. S2). Like in TbUBA1a and TcUBA1a, a Ser is present at the gatekeeper position. In contrast, LmUBA1b does not differ at the gatekeeper residue, but differs at the equivalents of human P554 and D579 (Fig. 7E, Supplementary Fig. S2). We investigated the TAK-243 inhibition of LmUBA1a and detected also for this protein a drastically reduced sensitivity to TAK-243 compared to hUBA1, consistent with the presence of a Ser at the gatekeeper position (Fig. 7F). The level of this resistance was lower than that of TbUBA1a, in agreement with the absence of a difference at the P554 positon of hUBA1. Most importantly, this observation shows that the possibility of selectively targeting UBA1s can be extended to *T. cruzi* and *Leishmania*.

## Discussion

We have studied the activity and inhibition of *T. brucei* UBA1s to determine whether the selective inhibition of these proteins over the human UBA1, is achievable and could be exploited for drug discovery. The results show that the TbUBA1s differ significantly from hUBA1 as they are resistant to the hUBA1 inhibitor TAK-243. We have pinpointed this difference to a specific pocket in the adenylation domain where the contact sites for the C_6_H_4_-SCF_3_ group of TAK-243 are located. Remarkably, both TbUBA1a and TbUBA1b differ in this pocket at the gatekeeper residue, the mutation of which was previously shown to impose TAK-243 resistance on hUBA1. Additionally, one other amino acid also contributes to the lack of inhibition by TAK-243. These data strongly support that differential targeting of TbUBAs and hUBA1 is possible. Moreover, we demonstrate that this is also applicable to *T. cruzi* and *Leishmania* UBA1s. Importantly, the results provide guidance towards the design of inhibitors with anti-trypanosome potential that should be focused on generating molecules that fit in the “gatekeeper pocket” of trypanosome, but not human, UBA1.

Although the genes for two *T. brucei* UBA1s were described previously^27^, the ubiquitin E1 activity of these gene products had not yet been demonstrated. We established that both proteins are functional (Fig. 1), and using structural modeling, that TbUBA1b contains a C-terminal UFD despite very low sequence homology in this region (Fig. 2). With the presence of two UBA1s that are only 24% identical to each other, the situation in *T. brucei* is different from that of other eukaryotes studied so far. However, our further BLAST searches showed that TbUBA1b orthologues are also present in other kinetoplastids, such as Strigomonas, Leptomonas, Phytomonas and Bodo, and that many other lower eukaryotes contain two distantly related UBA1 genes as well. Nevertheless, the significance of two UBA1 proteinss is not yet known. In T. brucei, both proteins are expressed at all life stages examined^35^ (see http://tritrypdb.org). Both proteins are important for *T. brucei* growth, although the knockdown of TbUBA1b had the more severe effect in a large-scale RNAi study^31^ (see http://tritrypdb.org). Out of ~10,000 genes tested, TbUBA1b ranked within the 1% of genes with the highest effect on *T. brucei* fitness, TbUBA1a ranked within the top 12%. Knockdown of TbUBA1b has also been shown to result in reduced protein ubiquitination^27^. Only one time point was studied (2 days after RNAi induction) so that the full extent of TbUBA1b’s involvement in overall ubiquitination is not yet clear. TbUBA1a and TbUBA1b may both be essential proteins with overlapping functions as is the case for UBA1 and UBA6^6,38^. These issues await further exploration, but it is clear that from a drug targeting perspective both *T. brucei* UBA1s are interesting candidates, although TbUBA1b may be the most attractive given its lower similarity to hUBA1 and its greater role in *T. brucei* fitness.

The lack of inhibition of both TbUBA1a and TbUBA1b by TAK-243 suggested that this compound has a very low affinity for the ATP-binding sites of these proteins. In contrast, the *T. brucei* UBA1s were twofold more sensitive to PYR-41 than hUBA1 (Fig. 3). We did not explore this further given the far greater differences with the more potent and more selective TAK-243. Although extensive resistance to TAK-243 was observed for both TbUBA1a and TbUBA1b, this was greatest for TbUBA1a (Fig. 3). This is consistent with our mutagenesis studies, which showed that in TbUBA1a two residues contribute equally to resistance, Q534 and S580. Only the mutation of both amino acids together led to a substantial increase in inhibition by TAK-243 (Fig. 5A,B). In contrast, in TbUBA1b, the mutation of T657 (equivalent to S580 in TbUBA1a) had a much greater effect than the mutation of S631 (equivalent to Q534 in TbUBA1a) (Fig. 5C,D). Although T657 of TbUBA1b was mutated together with the adjacent E656, the effect of these mutations can most likely be attributed primarily to T657, given the known role of the gatekeeper and the absence of an effect for a mutation of the E656 equivalent E559 in TbUBA1a (Fig. 5A,B) The presence of Q534 in TbUBA1a, and the equivalent S631 in TbUBA1b, instead of the proline at this position (P554) in hUBA1, are predicted to result in a wider pocket around the SCF_3_ group of TAK-243 (Fig. 6), leading to loss of contacts with this group. In TbUBA1a, the presence of an Ala (A533) instead of a Gly at the adjacent amino acid may further increase this effect. Our attempt to study whether a reciprocal mutation of P554 in hUBA1 would result in TAK-243 resistance was not successful because of insufficient yield and quality of proteins carrying a P554Q substitution.

The role of A580 in hUBA1 as a gatekeeper for adenosyl sulfamate inhibitors has been well established, and bulkier amino acids at this site such as a Ser (S560) in TbUBA1a and a Thr (T657) in TbUBA1b have been shown to interfere with inhibitor binding^19,37^. The gatekeeper was first defined in hUBA3, where mutations at A171 (the equivalent of A580 in hUBA1) were detected in cells with increased resistance to MLN4924^39,40^. An A580T substitution has also been introduced into recombinant hUBA1, which resulted in a six-fold decrease in inhibition by TAK-243^19^. More recently, a A580S mutation was identified in human cancer cell lines resistant to TAK-243^37^. Amino acid variations at the gatekeeper residue do not affect E1 activity as can be expected from its location outside the ATP-binding site^39,40^. An Ala at the gatekeeper position is highly evolutionary conserved in UBA1 (Fig. 1) and is also present in all other canonical E1s (UBA1, UBA2, UBA3, UBA6 and UBA7). However, the non-canonical E1s UBA4 and ATG7 contain a Thr and Ser at this position, respectively. Consistent with this, ATG7 is highly resistant to TAK-243 inhibition^18^. Our structural models of TbUBA1a and TbUBA1b show how a Ser or Thr at the gatekeeper position clash with the phenyl group of TAK-243 (Fig. 6).

The greater effect of the gatekeeper mutation T657A of TbUBA1b, compared to the S560A mutation of TbUBA1a, cannot be immediately explained by the structural models, which show a greater clash of TAK-243 with TbUBA1a than with TbUBA1b (Fig. 6). This stems from the presence of a Tyr in TbUBA1b (Y628), located opposite the gatekeeper residue, which appears to broaden the TAK-243 binding pocket. The fact that despite this, T657 of TbUBA1b interferes to a large extent with TAK-243 inhibition, most likely indicates that TAK-243 can only adopt a limited set of conformations all of which clash with the bulky T657 at the gatekeeper residue. It is further interesting to note that in the case of TbUBA1b the TAK-243 inhibition of the triple mutant was similar to that of hUBA1, whereas the triple mutant of TbUBA1a showed residual resistance. This also indicates that Y628 of TbUBA1b has little effect on TAK-243 resistance, while the above mentioned A533 of TbUBA1a may have a contributory effect.

In summary, the data presented here show that large differences exist between hUBA1 and *T. brucei* UBA1s at a region that is targeted by inhibitors. Novel molecules that bind to the TbUBA1s but not to hUBA1 may be generated by focusing on these differences at this “gatekeeper pocket”. The synthesis of TAK-243 derivatives in which the C_6_H_4_-SCF_3_ is replaced by other chemical groups may be a good starting point for this. For instance, molecules that can be accommodated in the presence of a Ser or Thr at the gatekeeper residue, can be expected to make fewer interactions with hUBA1 where the small Ala is present. Furthermore, it can be envisaged that longer or larger chemical groups are tolerated in the pocket of TbUBA1a and TbUBA1b due to the absence of the space-limiting Pro, P554 in hUBA1. Whether it will be possible to target both TbUBA1a and TbUBA1b with the same inhibitor, remains to be seen. Although the dual inhibition of these proteins will be favourable in view of efficiency and prevention of resistance, inhibition of the individual proteins is also predicted to result in *T. brucei* killing. Focusing on the more divergent TbUBA1b may be the most effective in this scenario. Not only is this protein less homologous to hUBA1 and does it appear to play a greater role in *T. brucei* fitness, it is also expected to accommodate a greater range of chemical groups than TbUBA1a based on the shapes of the “gatekeeper pockets” in our models, where space appears to be limited in TbUBA1a (Fig. 6). Crucially, we have also shown that the same strategy can be applied to obtain inhibitors against the *T. cruzi* and *Leishmania* UBA1s (Fig. 7).

To conclude, we have identified novel drug targets in trypanosomatids, the UBA1s, and have shown that these differ sufficiently from the human protein to be able to predict that trypanosome-selective inhibitors can be designed. The generation of such inhibitors may in the long-term lead to novel therapeutic agents. Although this path is still very long, the identification of novel promising drug targets in trypanosomes is an important first step towards this.

## Materials and Methods

### Cloning and cDNAs

The open reading frame for TbUBA1a was amplified by PCR from genomic DNA of *T. brucei* 927 (a kind gift of Mark Field, University of Dundee, Dundee, UK) using primers based on the gene sequence of Tb927.8.2640. PCR primers were designed for Gateway recombination cloning (ThermoFisher) and to introduce an N-terminal HRV 3C protease cleavage site. The PCR product was cloned into pDONR223 using BP clonase, and next into pDEST15 using LR clonase to generate an N-terminal GST fusion protein (plasmids and recombinases from ThermoFisher). The cDNA for TbUBA1b was amplified using primers complementary to gene Tb927.9.12650. Primers were designed for conventional cloning into EcoRI and NotI sites of pGEX4T3 (GE healthcare) to create an N-terminal GST fusion protein cleavable with thrombin. LmUBA1a was similarly cloned into EcoRI and NotI of pGEX4T3 and amplified from *Leishmania major* strain Friedlin genomic DNA (a kind gift of Maria Gomez, CSIC-UAM, Madrid, Spain) using primers based on gene LMJF_23_0550. All PCR reactions were performed using KOD high fidelity hotstart polymerase (Millipore). All cDNAs were verified by sequencing. A list of primers can be found in the supplementary information (Supplementary Fig. S10). The cDNA for human ubiquitin E1 as an N-terminal His-tagged fusion protein in pET21 (Millipore) was kindly provided by P. Brzovic (University of Washington, Seattle, USA). Human UbcH5a cloned into pGEX4T3 has been previously described^41^.

### Site-directed mutagenesis

Mutations were introduced into TbUBA1 cDNAs by PCR using KOD polymerase and complementary oligonucleotides (ThermoFisher) designed to change one or two amino acids at a time (for a list of oligonucelotides see Supplementary Fig. S10). Following removal of the parent plasmid by DpnI digestion, the PCR mix was transformed into *E. coli* strain GC5. Sequencing was used to verify the presence of the desired mutations. Sequential rounds of mutagenesis reactions were employed to generate triple mutants.

### Protein expression and purification

Fusion proteins were expressed in *E. coli* BL21DE3 (Novagen) grown in Terrific Broth, induced with 0.5 mM isopropyl β-D-1-thiogalactopyranoside (IPTG) at an OD600 of 0.7–1.0 and grown for another 16–20 hours at 16 °C. Cell pellets containing GST fusion proteins were re-suspended in 50 mM Tris pH 7.6, 150 mM NaCl, 1 mM DTT and protease inhibitors (EDTA-free protease inhibitor cocktail, Roche), sonicated 30 times for 10 sec and clarified by centrifugation at 18,000 × g and filtration through a 0.2 micron filter. Clarified lysates were loaded onto 1 ml HiTRAP GST columns (Generon) and eluted with 20 mM reduced glutathione in 50 mM Tris pH 7.6, 150 mM NaCl. Eluted fractions were incubated overnight at 4 °C with HRV 3 C (for TbUBA1a) or thrombin (for TbUBA1b and LmUBA1a) for cleavage from GST. Following concentration to 1 ml, samples were loaded onto a ProteoSEC. 6–600 size exlusion column (Generon) that was run in 50 mM Tris pH 7.6, 150 mM NaCl and peak fractions were collected. Peak fractions containing monomeric cleaved UBA1s were concentrated and frozen at −80 °C. For the purification of hUBA1 with a non-cleavable His-tag, cell pellets were re-suspended in 20 mM Tris pH 7.6, 0.5 M NaCl, 40 mM Imidazole, 1 mM DTT and protease inhibitors. Following sonication and clarification as described above, lysates were loaded onto 1 ml Ni/NTA columns and eluted with an Imidazole step gradient. Fractions containing the hUBA1 were concentrated before further purification on a ProteoSEC. 6–600 size exlusion column (Generon) as described above. Protein purity was determined by SDS-PAGE and Coomassie staining, followed by analysis on ImageQuant LAS 500 (GE healthcare). Protein concentration was measured by NanoDrop 2000 (ThermoFisher).

### *In vitro* E1-E2 transthioesterification assay, inhibitors

Each 10 μl of E1-E2 trans-thioesterifcation reactions contained 0.25 μg UBA1 (0.2 μM final concentration), 1.7 μg UbcH5a (10 μM final), 3.5 μg human ubiquitin (25 μM final), 100 μM Mg~ATP in 20 mM Tris pH 7.6, 150 mM NaCl. UBA1s and UbcH5a were purified as described above, ubiquitin was from Enzo Life Sciences and chemicals from Sigma-Aldrich. Reactions were run at 25 °C in a heating block. For time courses, one master mix was prepared and 10 μl samples were taken from this at the indicated times. Time zero was taken before the addition of Mg~ATP (from a 2 mM solution) to start the reaction. To stop the reactions, 5x non-reducing SDS-PAGE loading buffer was added followed by immediate heating at 95 °C. For inhibitor assays, master mixes without Mg~ATP were prepared. 9 μl of this mix was added to tubes containing either 0.5 μl DMSO or different concentrations of the inhibitor, followed by a 30 min incubation at 25 °C. Reactions were then started by the addition of 0.5 μl Mg~ATP added from a 2 mM stock and allowed to proceed for 30 min at 25 °C. Reactions were stopped as described above. TAK-243 (Chemietek) was prepared as a 10 mM stock in DMSO, and Pyr-41 (Sigma) as a 50 mM stock in DMSO. All inhibitor dilutions were made in DMSO.

### Analysis and quantification of E1-E2 transthioesterification assays

For analysis, equal volumes of transthioesterification reactions were loaded onto 15% non-reducing SDS-PAGE gels alongside prestained protein ladders (PageRuler, ThermoScientific), followed by Coomassie staining. Gel images were captured with ImageQuant LAS500 and analyzed with ImageQuant TL8.1 software. Band intensities of UbcH5a (E2) and UbcH5a~Ub (E2~Ub) were determined following background subtraction (rolling ball method). For each lane, the proportion of E2~Ub relative to the total amount of E2 (E2 + E2~Ub) was determined. For time courses, the E2/(E2 + E2~Ub) ratio at 30 min was set at 100% for representation in graphs. For inhibitor assays, the E2/(E2 + E2~Ub) ratio in the absence of inhibitor was set at 100% for representation in graphs. All experiments were performed at least two times. IC50s were calculated using the web-based tool https://www.aatbio.com/tools/ic50-calculator.

### Structural modelling

For tertiary structure prediction of TbUBA1a and TbUBA1b, the Phyre2^32^ protein fold recognition server was used in intensive mode. Analysis of structures and the generation of images were performed using UCSF Chimera^42^.

## Supplementary information

Supplementary Information

## Data Availability

The datasets generated during and/or analysed during the current study are available from the corresponding author on reasonable request.

## References

[1] Stuart K (2008). Kinetoplastids: related protozoan pathogens, different diseases. J Clin Invest.

[2] Bilbe G (2015). Infectious diseases. Overcoming neglect of kinetoplastid diseases. Science.

[3] Finley D, Ciechanover A, Varshavsky A (1984). Thermolability of ubiquitin-activating enzyme from the mammalian cell cycle mutant ts85. Cell.

[4] Kulkarni M, Smith HE (2008). E1 ubiquitin-activating enzyme UBA-1 plays multiple roles throughout C. elegans development. PLoS Genet.

[5] McGrath JP, Jentsch S, Varshavsky A (1991). UBA 1: an essential yeast gene encoding ubiquitin-activating enzyme. EMBO J.

[6] Schulman BA, Harper JW (2009). Ubiquitin-like protein activation by E1 enzymes: the apex for downstream signalling pathways. Nat Rev Mol Cell Biol.

[7] Haas AL, Rose IA (1982). The mechanism of ubiquitin activating enzyme. A kinetic and equilibrium analysis. J Biol Chem.

[8] Cappadocia L, Lima CD (2018). Ubiquitin-like Protein Conjugation: Structures, Chemistry, and Mechanism. Chem Rev.

[9] Pickart CM, Eddins MJ (2004). Ubiquitin: structures, functions, mechanisms. Biochim Biophys Acta.

[10] Komander D, Rape M (2012). The ubiquitin code. Annu Rev Biochem.

[11] Lake MW, Wuebbens MM, Rajagopalan KV, Schindelin H (2001). Mechanism of ubiquitin activation revealed by the structure of a bacterial MoeB-MoaD complex. Nature.

[12] Lee I, Schindelin H (2008). Structural insights into E1-catalyzed ubiquitin activation and transfer to conjugating enzymes. Cell.

[13] Olsen SK, Lima CD (2013). Structure of a ubiquitin E1-E2 complex: insights to E1-E2 thioester transfer. Mol Cell.

[14] Haas AL, Warms JV, Hershko A, Rose IA (1982). Ubiquitin-activating enzyme. Mechanism and role in protein-ubiquitin conjugation. J Biol Chem.

[15] Field-Smith A, Morgan GJ, Davies FE (2006). Bortezomib (Velcadetrade mark) in the Treatment of Multiple Myeloma. Ther Clin Risk Manag.

[16] Xu GW (2010). The ubiquitin-activating enzyme E1 as a therapeutic target for the treatment of leukemia and multiple myeloma. Blood.

[17] Yang Y (2007). Inhibitors of ubiquitin-activating enzyme (E1), a new class of potential cancer therapeutics. Cancer Res.

[18] Hyer ML (2018). A small-molecule inhibitor of the ubiquitin activating enzyme for cancer treatment. Nat Med.

[19] Misra M (2017). Dissecting the Specificity of Adenosyl Sulfamate Inhibitors Targeting the Ubiquitin-Activating Enzyme. Structure.

[20] Brownell JE (2010). Substrate-assisted inhibition of ubiquitin-like protein-activating enzymes: the NEDD8 E1 inhibitor MLN4924 forms a NEDD8-AMP mimetic *in situ*. Mol Cell.

[21] Cardoso J (2008). Inhibition of proteasome activity blocks Trypanosoma cruzi growth and metacyclogenesis. Parasitol Res.

[22] Muñoz C, San Francisco J, Gutiérrez B, González J (2015). Role of the Ubiquitin-Proteasome Systems in the Biology and Virulence of Protozoan Parasites. Biomed Res Int.

[23] Ponder EL, Bogyo M (2007). Ubiquitin-like modifiers and their deconjugating enzymes in medically important parasitic protozoa. Eukaryot Cell.

[24] de Diego JL (2001). The ubiquitin-proteasome pathway plays an essential role in proteolysis during Trypanosoma cruzi remodeling. Biochemistry.

[25] Cerqueira PG (2017). Effect of ionizing radiation exposure on Trypanosoma cruzi ubiquitin-proteasome system. Mol Biochem Parasitol.

[26] Khare S (2016). Proteasome inhibition for treatment of leishmaniasis, Chagas disease and sleeping sickness. Nature.

[27] Chung WL, Leung KF, Carrington M, Field MC (2008). Ubiquitylation is required for degradation of transmembrane surface proteins in trypanosomes. Traffic.

[28] Kumar D, Saha S (2015). HAT3-mediated acetylation of PCNA precedes PCNA monoubiquitination following exposure to UV radiation in Leishmania donovani. Nucleic Acids Res.

[29] Vince JE, Tull D, Landfear S, McConville MJ (2011). Lysosomal degradation of Leishmania hexose and inositol transporters is regulated in a stage-, nutrient- and ubiquitin-dependent manner. Int J Parasitol.

[30] Zoltner M, Leung KF, Alsford S, Horn D, Field MC (2015). Modulation of the Surface Proteome through Multiple Ubiquitylation Pathways in African Trypanosomes. PLoS Pathog.

[31] Alsford S (2011). High-throughput phenotyping using parallel sequencing of RNA interference targets in the African trypanosome. Genome Res.

[32] Kelley LA, Mezulis S, Yates CM, Wass MN, Sternberg MJ (2015). The Phyre2 web portal for protein modeling, prediction and analysis. Nat Protoc.

[33] Lv Z, Williams KM, Yuan L, Atkison JH, Olsen SK (2018). Crystal structure of a human ubiquitin E1-ubiquitin complex reveals conserved functional elements essential for activity. J Biol Chem.

[34] Lv Z (2017). S. pombe Uba1-Ubc15 Structure Reveals a Novel Regulatory Mechanism of Ubiquitin E2 Activity. Mol Cell.

[35] Siegel TN, Hekstra DR, Wang X, Dewell S, Cross GA (2010). Genome-wide analysis of mRNA abundance in two life-cycle stages of Trypanosoma brucei and identification of splicing and polyadenylation sites. Nucleic Acids Res.

[36] Ungermannova D (2012). Identification and mechanistic studies of a novel ubiquitin E1 inhibitor. J Biomol Screen.

[37] Barghout, S. H. *et al*. Preclinical evaluation of the selective small-molecule UBA1 inhibitor, TAK-243, in acute myeloid leukemia. *Leukemia* (2018). 10.1038/s41375-018-0167-0 29884901

[38] Liu X (2017). Orthogonal ubiquitin transfer identifies ubiquitination substrates under differential control by the two ubiquitin activating enzymes. Nat Commun.

[39] Milhollen MA (2012). Treatment-emergent mutations in NAEβ confer resistance to the NEDD8-activating enzyme inhibitor MLN4924. Cancer Cell.

[40] Toth JI, Yang L, Dahl R, Petroski MD (2012). A gatekeeper residue for NEDD8-activating enzyme inhibition by MLN4924. Cell Rep.

[41] Bijlmakers MJ (2016). A C2HC zinc finger is essential for the RING-E2 interaction of the ubiquitin ligase RNF125. Sci Rep.

[42] Pettersen EF (2004). UCSF Chimera–a visualization system for exploratory research and analysis. J Comput Chem.

