# Supplementary material for: Differential Inhibition of Human and Trypanosome Ubiquitin E1S by TAK-243 Offers Possibilities for Parasite Selective Inhibitors
Source: Sci Rep. 2019 Nov 7;9:16195. doi: 10.1038/s41598-019-52618-3 (PMC6838199; doi:10.1038/s41598-019-52618-3)

SUPPLEMENTARY INFORMATION

Manuscript:

"Differential inhibition of human and trypanosome ubiquitin E1s by TAK-243 offers possibilities for parasite selective inhibitors."

D. Roeland Boer and Marie-José Bijlmakers*

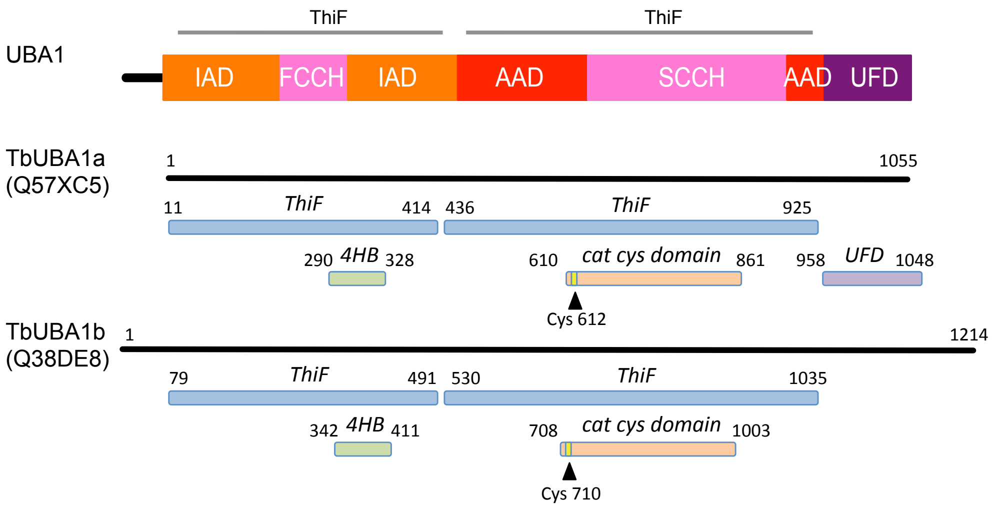

Figure S1. Domains in TbUBA1a and TbUBA1b as found on the UniProt website.

The domains are: the THIF-type NAD/FAD binding fold (ThiF, pf00899), the 4 helix bundle (4HB, pf16191), the UBA-E1-thiolCys (catalytic cys domain, pf10585) and the E1-Ubiquitin fold domain (UFD, pf09358).

Figure S2

```
P22314|hUBA1_          1 -----------------MSSSPLSKKRRVSGPDPKPGSNCSPAQSVLSEVPSVPTNGMAK
Q57XC5|TbUBA1a         1 ------------------------------------------------------------
Q38DE8|TbUBA1b         1 MPKHQRDRFSNCPAFCSHTSNKLFRTELTHY-SSDTTR--NVNINGFTKMSKMAGATADS
Q4DYM1|TcUBA1a         1 ------------------------------------------------------------
EKG03124.1 TcUBA1b     1 MRKHTRNHESDHYALYSK-NKKFYRTGTSQGFNCNKNKVDTAFVNAETEAAEMSVVEAIA
Q9NF77|LmUBA1a         1 ------------------------------------------------------------
E9AFD0|LmUBA1b         1 ----------------------------------------------------------ME

P22314|hUBA1_         44 NGSEADIDEGL---YSRQLYVLGHEAMKRLQTSSVLVSGLRGLGVEIAKNIILGGVKAVT
Q57XC5|TbUBA1a         1 -MTSEEQRRQL---YNRQEYVVGTETQAKYGCTDVLVVGACGLGAEIIKNLTLTGVRSIK
Q38DE8|TbUBA1b        58 SDASSAIDSKFLDQQSRTIGTYGLETMAKLISFKVLVVGCGGVGIETAKNLALAGVHTIT
Q4DYM1|TcUBA1a         1 -MKHEEEKRQL---YNRQEYVVGVETQAKYGSTDVLVVGACGLGAEIVKNLALTGVRSIK
EKG03124.1 TcUBA1b    60 APAASAIDSKFLDKQSRTIGTYGLETMVKLISFKVLIVGCGGVGIEAAKNLSMAGVHTII
Q9NF77|LmUBA1a         1 MLSEEEQKRQL---YSRQEYVVGSETQAKYGSTHVLVVGATGLSAEIIKNVVLTGVKSVK
E9AFD0|LmUBA1b         3 AERKALIDQRYLDKQSRTIGTYGLETMAKLIAFKVIIVGCGGVGIEIAKNLALAGIHTIR

P22314|hUBA1         101 LHDQGTAQWADLSSQFYLREEDI--GKNRAEVSQPRLAELNSYVPVTAYTGPLVEDFLSG
Q57XC5|TbUBA1a        57 VLDNGLATLQDLGTNFFLTPADM--GKPRAEVVAARAQELNRFVSVTAVDVP-LHEVIPA
Q38DE8|TbUBA1b       118 LCDPKKAELKDMGVNFAVTETTIKAGLTRAEASKRLVAELNPNVRVRTVDAI-DEAVVSE
Q4DYM1|TcUBA1a        57 IMDSGTAILQDLGTNFFLTPQDV--GQPRADVVARRAQELNRFVHITAVTSP-LHEVIPD
EKG03124.1TcUBA1b    120 LCDPAKAQPKDMGVNFAVTEAAVRSGLTRAEASQRLVSELNPNVRVRVVDAL-SEAVVSQ
Q9NF77|LmUBA1a        58 VLDDAVVTIEDLGTNFFLRPDDV--GKARGAAVAQAAKELNRFVEVSSVSGD-PLLHIPA
E9AFD0|LmUBA1b        63 FYDPRKPTVQDMGVNFAVTPQSMASGKTMAELSAAYISELNPNTRVGVLAEL-TTATVAD

P22314|hUBA1         159 FQVVVLTNT----PLEDQLRVGEFCHNRG--IKLVVADTRGLFGQLFCDFGEEMILTDSN
Q57XC5|TbUBA1a       114 VHVVVFVNQ----RTTLLLAENAMARKHN--VKFVACESRGVAGCVFVDAGPSFTVLDPD
Q38DE8|TbUBA1b       177 VNCVVYTSAAADWSSKTLLKWDQFCRTRTPAISFIFAFQGGSLASVFADHAPNFTVKDAD
Q4DYM1|TcUBA1a       114 VHVVVFVNQ----RTTALVGENALARKHD--VKFVACESRGIVGCVFVDAGPSFSVLDPD
EKG03124.1TcUBA1b    179 VNALVFTSAAPDYSLRTLKKWNKFCHDHSSPISFIFAFQGGALGSVFADHGAHFTVKDPD
Q9NF77|LmUBA1a       115 VHVVIYTNA----YTSTLAAANKVARENK--VKFISCESRGVCGCIFVDGGESLDIVDTD
E9AFD0|LmUBA1b       122 NVALIFTTAAPDLRLTTLSEWNAFCHNHTPAISFVLALQTGTMGSVFTDHGPSFVVKDAD

P22314|hUBA1         213 GEQPLSAMVSMVTKDNPGVVTCLDEARHGFESGD---------FVSFSEVQGMVELNGN-
Q57XC5|TbUBA1a       168 GEETVVCVVTNIS--RDGVVSLHEDKKHECEVGG---------RVFLTGLVSPESLNSTV
Q38DE8|TbUBA1b       237 GRPMLQKLIVEVLTKRDKSGVEYTRVRYETPEGQTPGAFRDYTEVKFSEVKGLCKANGE-
Q4DYM1|TcUBA1a       168 GEETVSCIVTSVS--RDGVVSLHEDKKHECEVGS---------HIFFTGVVSPAAVNADI
EKG03124.1TcUBA1b    239 GRPMLQKSIVEVITKQDKTGTAYTRIRYETPEGQTPGALRDYTRFKFTEVRGLCKANGE-
Q9NF77|LmUBA1a       169 GEDTVTCVVTAMS--SDGLVTLHEEKNHECEIGS---------KVYFTGLTELPQANTTE
E9AFD0|LmUBA1b       182 GRPMLQKLITEVVTLRDKTGEMYTRIRYETPEGQTPGALRDYTQIKLSEVQGLLKPDGT-

P22314|hUBA1         263 --------------------QPM---------EI--KVLGPYTFSICD---TSNF-SDYI
Q57XC5|TbUBA1a       217 DPFALHNGRATTECAQGDNSPTGASSSLRLFEVS--EVVSPFHLRLKDFGAIVGD-SPIE
Q38DE8|TbUBA1b       296 --------------------SIN----GNVFKGVVCTGDPPNTVRIYPSLESQGY-SAYE
Q4DYM1|TcUBA1a       217 DPSTIHG-RC----------QSAASPLLKLFEVA--EVISPFILRLKDFEAAVGN-SPIE
EKG03124.1TcUBA1b    298 --------------------SVN----DNIFNGVMCPNDPRDTVRIYPSLESQGY-SAYE
Q9NF77|LmUBA1a       218 ---------------------PATPSAWKLFEVA--EVISPHTMRLKGVSELVSAGTIIH
E9AFD0|LmUBA1b       241 --------------------SAN----GQVYDGVICPSDPRDTVRVYPAFETQGY-SPYE

P22314|hUBA1         288 --RGGIVSQVKVPKKISFKSLVASLAEP-DFVV-TDFAKFSRPAQLHIGFQALHQFCAQH
Q57XC5|TbUBA1a       274 TGYACYLHTTKRKVLVGFKDLQLSVMQP-EFVT---LFDSEKKMMAPMTLHALFRAVHSH
Q38DE8|TbUBA1b       331 --TAGFLHEMKESQQLKFRALSEALSCPGQFVPVSSMMDGSEESQSHLTFTALLRFFDKH
Q4DYM1|TcUBA1a       263 VGTGAYLHTTKRHVLMGFKDLEQSVADP-TFVS---IFDSEEKVNAPATLHALFRALHDH
EKG03124.1TcUBA1b    333 --TGGFLHELKEVFQIEFRTLEEAVVCPGRFVPVSPMMDGSEESQSHLALHALLNFLDRH
Q9NF77|LmUBA1a       255 VGTSAYLHTTKKGRREHYRTLGECLDNP-ECLM---IFDKEEKYTAATTLHAMFTAVARH
E9AFD0|LmUBA1b       276 --TGGFLHELKEVKVLPFRPLSEALAAPGPFVSVSPMMDNSEESLTHVTLHALLRYADAH
```

Figure S2 continued

```
P22314|hUBA1         344 -GRPPRPRNEEDAAELVALAQAVNARALP--A----------------------------
Q57XC5|TbUBA1a       330 -GKLPT--TPIEVRDVLKAAEAYFSSGND--Q----------------------------
Q38DE8|TbUBA1b       389 G-RLPELHNLSEANEVVSLAKAVNEENKAADAKLEKVDHPMFLQHENKEFPSRLAPPPPP
Q4DYM1|TcUBA1a       319 -GTLPT--TPTEVNSLLNLAEAYHSSCNS-------------------------------
EKG03124.1TcUBA1b    391 G-RPPKLHDVSEAEEALSIAKEINIENKRRS-KANTETYQMFLEPENEEFPARLAPSPPP
Q9NF77|LmUBA1a       311 -GCAPT--SPAEVEAVVKAAQAINPNA---------------------------------
E9AFD0|LmUBA1b       334 AGKLPELHNAAQATAVVELAKKVLEENKAMPAPPEQRTTG---KPSNAEFPYKVPPPPPP

P22314|hUBA1         373 --VQQNNLDEDLIRKLAYVAAGDLAPINAFIGGLAAQEVMKACSGKFMPIMQWLYFDALE
Q57XC5|TbUBA1a       357 ---VHNGFDVETAESILSVMHGRLNPMDCFIGGLASQEVLKVCSGKFTPLRQWLYYDARE
Q38DE8|TbUBA1b       448 TPLCVETLDEGFVCSQALVSAAELQPLCAVWGAVLAQEIVKIT-GKYTPICQWLHVGYSS
Q4DYM1|TcUBA1a       345 -----GHLDVEFSKKALSVIHGNLNPMACLIAGIASQEVLKVCSGKFTPIQQWLYYDARE
EKG03124.1TcUBA1b    449 VPLTVDEVDESFIRTQSLVADAELQPLCAFFGAVVAQEIVKIT-GKYTPICQWFHFRCDA
Q9NF77|LmUBA1a       335 --------EATVMRTLLPVFGGDLNPMACFIGGMAAQEALKVCSGKFTPLHQWVYYDARE
E9AFD0|LmUBA1b       391 APLVLESLDEKAVMAEALVARAELQPLASFFGAVVAQEIVKIT-GKYSPIHQWFHLSCAA

P22314|hUBA1         431 CLPEDKEVL-T--------EDKCL-QR--QN-RYDGQVAVFGSDLQEKLGKQKYFLVGAG
Q57XC5|TbUBA1a       414 LLVARGEMS-E--------T-GCVSTA-PGGSRYDGQIAVLGSSFQSFLSRQRVFIVGAG
Q38DE8|TbUBA1b       507 ILASNASYT-K--------SPQEY-KV--VDHRYKHLISLFGKTFVEKLNNLKLFMVGCG
Q4DYM1|TcUBA1a       400 LLVARGEVA-E--------A-DLRPPS-PTGSRYDKQIAVLGAAFQSYLSKQRAFIIGAG
EKG03124.1TcUBA1b    508 ILASSAMYT-S--------SG-DY-KP--TNSRYDHLIALLGKNFQKKLESLRVFMVGCG
Q9NF77|LmUBA1a       387 VLQVWQYGAKTVSASTLSSSAAVFPDAPAARSRYAGQEAVLGHAFQEYLRQQKAFIVGAG
E9AFD0|LmUBA1b       450 VRPERADYS-S--------E--EF-RP--MNSRYDHIISIFGKGFQQLLQNLRLFMVGCG

P22314|hUBA1         478 AIGCELLKNFAMIGLGCGEGGEIIVTDMDTIEKSNLNRQFLFRPWDVTKLKSDTAAAAVR
Q57XC5|TbUBA1a       463 ALGCELIKNVACMGF----G-AVSITDMDTIEMSNLSRQFLFRNSHIGQQKSKVAGEAAR
Q38DE8|TbUBA1b       555 ALGCENIKNFALCGMSCGPRGSFVVTDNDRIEVSNLSRQFLFREENVGQPKSAVAVSRMK
Q4DYM1|TcUBA1a       449 ALGCELIKNAACMGF----G-GISITDMDSIEISNLSRQFLFRNSHIGQHKSRVAGEAAM
EKG03124.1TcUBA1b    555 ALGCENIKNFALCGVACGPNGSLLVTDNDRIEVSNLSRQFLFREENVGQPKSVAAAARMR
Q9NF77|LmUBA1a       447 ALGCELIKNVALMGF----G-EVSITDMDTIEMSNLSRQFLFRNHHIGRPKSVVAAEAAG
E9AFD0|LmUBA1b       496 ALGCENVKNFALCGITCGTGGSLVVTDNDRIEVSNLSRQFLFREENVGQSKSAAATARMR

P22314|hUBA1         538 QMNPHIRVTSHQNRVGPDTERIYDDDFFQNLDGVANALDNVDARMYMDRRCVYYRKPLLE
Q57XC5|TbUBA1a       518 AINGDLKVSAYLEKVAQETENVFDEKFWESHSLVLNALDNVESRKYVDARCLFFRKPLFE
Q38DE8|TbUBA1b       615 SINKDVKADARQDYVGSNTEHIYHDVFWNGLDAVVNALDNMETRLYVDQKCVNFHKILVE
Q4DYM1|TcUBA1a       504 AINHDLHVTSFVEKVSVETEGIFNEAFWDSHAVVLNALDNVQSRKYVDSRCLFYKKPLFE
EKG03124.1TcUBA1b    615 IMNKDVAIDPRQDYVGATTEHLYHDIFWDGLDVVVNALDNMETRLYVDQQCVKFQKILVE
Q9NF77|LmUBA1a       502 HINADVKITAYEAKMGPETEAIFNEDFWVQQAVILNALDNVMSRKYVDSRCLFYQKPLLE
E9AFD0|LmUBA1b       556 QMNPDANVDARQDFIGTTTEHLYPDTFWQSLNVVVNALDNIEARLYVDQQCVRFQKVLVE

                                                   *
P22314|hUBA1         598 SGTLGTKGNVQVVIPFLTESYSSSQDPPEK-SIPICTLKNFPNAIEHTLQWARDEFEGLF
Q57XC5|TbUBA1a       578 SGTLGPKCNVQCVIPYCTESYSSSYDPPEK-SIPLCTLKNFPNVIEHTIQWARDNFDAVF
Q38DE8|TbUBA1b       675 AGTMGTGGNVDIVVPGKTTSYSDGGAADTTGGIPMCTLRNFPYTSDHCTEWARAQFDDLF
Q4DYM1|TcUBA1a       564 SGTLGTKCNVQCIIPYCTESYSSSHDPPEK-AIPLCTLKNFPNAIEHTIQWARDNFHVLF
EKG03124.1TcUBA1b    675 AGTMGTGGNVDIIVPGKTTSYADGGAADASGGIPMCTLRNFPYIFDHCIEWSRAQFDDLF
Q9NF77|LmUBA1a       562 SGTLGTKCNMQPAIPFVTESYSSSYDPPEK-GIPLCTLKNFPNAIEHTIQWARDLFHLLF
E9AFD0|LmUBA1b       616 AGTMGTGGNVDIIVPGRTSSYADGGAADQTGGIPMCTLRNFPYIYDHCIEWARAQFDDLF

P22314|hUBA1         657 KQPAENVNQYLTDPK-FVERTLRLAGTQPL------------EVLEAVQRSL-VLQRPQT
Q57XC5|TbUBA1a       637 FSTPSDVNGYLEDPTTFASNLERDPGTKSI-------------VLKAVRDAL--VQWPKD
Q38DE8|TbUBA1b       735 VSPMQTVRQLLENPAAFTERIKNEVNNAQSAGERLSLVEKNLGILQGIQKTVTTLSAGVS
Q4DYM1|TcUBA1a       623 TNTPEEVNSYLQDPTTFAANLERDPATKTM-------------ALKAVRDAL--LRWPMD
EKG03124.1TcUBA1b    735 VFPMQTVEQLVEDPTAFKARIEREINAAQSSGERLSLVEKHLGILHPLQKVLSNLSSGVN
Q9NF77|LmUBA1a       621 VSVPADVNQYLNDPVAFANSLRNDPAAADA-------------ALQNVNDAL--SRWPQN
E9AFD0|LmUBA1b       676 VSPMQAAQQIIEDPAAFTQRIHHEVSSGSSAGERRSLIDKNVGPLKLLKRTLTILADGPT
```

Figure S2 continued

```
P22314|hUBA1          703  WADCVTWACHHWHTQYSNNIRQLLHNFPPDQLTSSGAPFWSGPKRCPHPLTFDV----NN
Q57XC5|TbUBA1a        682  AADCVRMARSLFHEYFNSSFRQLLHNLPLDKRNDNGDLFWSGAKKPPKPQEFSV----DS
Q38DE8|TbUBA1b        795  MEKCVQCAWETMFHLFRDRILDLQRSFPKDAKKKNGEKFWSGHRKYPTPLEVNIKALSSD
Q4DYM1|TcUBA1a        668  AADCVRIARRLYHEYFSDAFRQLLYNIPIDKRNENGELFWSGAKKPPTPQEFSP----DS
EKG03124.1TcUBA1b     795  MEKCFQCAWELMFYLFRDRIMDLQRSFPRDAKKKNGEDFWSGHRKYPTALNVDPKTIASN
Q9NF77|LmUBA1a        666  EQNCVRLARLLYQEHFNDGFRQLLHSIPLDKRNEDGQLFWGGAKKPPTPQEFDV----NS
E9AFD0|LmUBA1b        736  MDRCVALGWEQLFKMFRDRILDLQAAFPRGAKKKNGEDFWSGHRKYPTALQVTAADIATN

P22314|hUBA1          759  PLHLDYVMAAANLFAQTYGLTG--SQ---------------DRAAVATFLQSVQVPEFTP
Q57XC5|TbUBA1a        738  ELNVSFVYHCAKLLAQVYNLSAF-TL---------------SVKEVAELAMQVAVPGFVP
Q38DE8|TbUBA1b        855  PDVVEFLISAANLFACMYGIHPQKHEPRLNDPKKRWMQQYRTLDWLNGVMKNCTVPEYKP
Q4DYM1|TcUBA1a        724  ELSMSFVYHCAYLLACVYGLPPF-TL---------------SRADVARVAGETSVPEFVP
EKG03124.1TcUBA1b     855  KDAVEFLIAASNLFACMYGVHPPKHEPRFNDANNRWMQQYRSLEWINKIIEKREVPVYHP
Q9NF77|LmUBA1a        722  EQDTEFVYHCACLFAKVYQLPAF-SL---------------SKEETARLAAAVTVPDFVP
E9AFD0|LmUBA1b        796  PDAKNFLVAAINLYACMFGVHPPKHEARFNDEKNRWMQEYRTDAWIQAEVNKLPTPAYVA

P22314|hUBA1          802  KS------GVKIHVSDQELQSANASVDDSRLEELKATLPSPDKLPGFKMYPIDFEKDDDS
Q57XC5|TbUBA1a        782  RE---------ARFETNEAENKEGAAAQLVGDLTMQDLPPVSQFNSRRMNPLVFEKDDPN
Q38DE8|TbUBA1b        915  GSVEGLDDDLLQSMEKQEVSKDETTKEQTLNNLLSSVVALAQKCHNMNTVPLDFEKDDDD
Q4DYM1|TcUBA1a        768  RQ---------AVFATSEAEK-EESVANLAAEIGLQDLPPVSEFHGRRMVPEFFEKDDPT
EKG03124.1TcUBA1b     915  GAVEGLDDDILDAIQTHDGAKKEETKEEQLGQLLCNIMTLAGSCRGTKATPLDFEKDDDD
Q9NF77|LmUBA1a        766  RH---------AVFATSESQTSQQTSSS--RGLTVEQLPPVAHFGSRRMRAEEFDKDDIT
E9AFD0|LmUBA1b        856  GSVDNLDDDLAADAQEGKQVSMEESEAE-LQGLLADVAALASKCKGSKAAALEFEKDDDD

P22314|hUBA1          856  NFHMDFIVAASNLRAENYDIPSADRHKSKLIAGKIIPAIATTTAAVVGLVCLELYKVVQG
Q57XC5|TbUBA1a        833  NSHMDYITACSNLRATAYSIPPADVHYTKRIAGRIIPAMVTTTALVTGLVGIEALKYLLL
Q38DE8|TbUBA1b        975  NFHIDFVAATSNLRARNYDIPTQDRFKVKLVAGKIIPAIATTTAAVTGLALIEYFKALLS
Q4DYM1|TcUBA1a        818  NHHVEYITACSNMRAVAYNIPPADVHHTKRIAGKIIPAMVTTTALVTGLVGLEVLKRLLM
EKG03124.1TcUBA1b     975  NFHIDFVTAASNLRASNYDIPTQDRMKVKLVAGKIIPAIATTTSAVTGLALIEYFKALQG
Q9NF77|LmUBA1a        815  NHHVQFITYCSNLRARAYSIPVADFNQTKRIAGNIIPAMVTTTSLVTGLVGFEMLKYLLI
E9AFD0|LmUBA1b        915  NFQIDFVAAASNLRAENYGIPTQDRLKVKLVAGKIIPAIATTTSAVTGLGLIELFKVLQN

P22314|hUBA1          916  HRQ----------------------------LDSYKNGFLNLALPFFGFSEPLAA--PRH
Q57XC5|TbUBA1a        893  AHRENGAQGLAKANP-----IT--EKVQEEYLSLYRNAFVNVALPFMAFSDPIAA--PAK
Q38DE8|TbUBA1b        1035 NDVSCLRNGMLDIGT-----NN-YVLFERD-------------APLKHRTRVDKTYLPEQ
Q4DYM1|TcUBA1a        878  TQRQERSGMPANAVP-----TY--EEIQKQ-LSIYRNAFVNIALPFIAFSDPIIA--SGA
EKG03124.1TcUBA1b     1035 NDISCLRNGMIDVGT-----NN-YVLFERD-------------APIKNRTKIVSTYLPEQ
Q9NF77|LmUBA1a        875  QFHHARKPAVNGTGSSHGNFYLDADEEPEKLVTLFRSAFVNIALPFIAFSDPIIA--PSH
E9AFD0|LmUBA1b        975  KDISVLRNGMLDVGT-----NN-YVLFERD-------------LPIKNFTKVVATYIPEQ

P22314|hUBA1          946  QYY------------NQEWTLWDRFEVQGLQPNGEEMTLKQFLDYFKTE----------H
Q57XC5|TbUBA1a        944  TVP-------MPDGSSVRWGIWDRIDIN----EGRDITVKELVSILEKR----------H
Q38DE8|TbUBA1b        1076 DYTYKKKVICLPEGY----TKYDMIEVP----ITKATTVQQFATELEKKLNTLLPTGMNA
Q4DYM1|TcUBA1a        928  TYP-------LPDGTSVRWGIWDRIDVN----EGRDLTVQELVTVLESR----------Y
EKG03124.1TcUBA1b     1076 DYTYKKKLIRVPDGF----TKYDSIDVP----ITIHTTVQQFATMLENQLNALLPAGTEG
Q9NF77|LmUBA1a        933  SYA-------LPSGKKLRWGIWDRIDVS----EGRDMLVKELVQLLHDR----------Y
E9AFD0|LmUBA1b        1016 DYTYKKKIIRVPEGF----TKYDMIRIP----VTPATTVKAFAAALEAVLNKTLPEGADY

P22314|hUBA1          984  KLEITMLSQGV-SMLYSFFMPAAKLKERLDQPMTEIVS-RVS--KR-----KLGRHVRAL
Q57XC5|TbUBA1a        983  QLEIFIIALPCGKMVYSQFG-NVKDR---DKPVSVVVREKTK-GEE-----KDELSCICF
Q38DE8|TbUBA1b        1128 GCEVSAIGVGKG-SLWNGLPKHANTNCSLMDIIEKQKL-SEAGGKLPRPFWENRTHFHDL
Q4DYM1|TcUBA1a        967  QVELFIIALASGKIIYSQFG-NTKDR---GKPVSTVVLEKGE--QL-----QDGEDCCCL
EKG03124.1TcUBA1b     1128 SCEIVGIGVGHG-MLWNGSKKHANTNLSLMQLIEQQKM-TEAGGKLSQPFWQNRTQFCEL
Q9NF77|LmUBA1a        972  ELEVFMIALKNGKMIYTEFGGKAKDK---EKRVSEVAQDKGE--KV-----QDGIDYFDL
E9AFD0|LmUBA1b        1068 AYEVDGLGVGKG-MLWNGRSSHANTNASLMKVIEQQKA-SEAGGTLPAPFWQNRFQFCDL
```

```
P22314|hUBA1        1035 VLELCCNDESG----EDVEVPYVRYTI--R
Q57XC5|TbUBA1a      1033 VATGSIGDN-------DVDIPLIYYRYKDF
Q38DE8|TbUBA1b      1186 SVTVSIDDDDANVDEVDVETATILLRIQQ-
Q4DYM1|TcUBA1a      1016 VATGSISDV-------DVDIPVIRYRFRNF
EKG03124.1TcUBA1b   1186 SVTVSLDDGDTSVDEADVETAMIRLRITQ-
Q9NF77|LmUBA1a      1022 VVTGMIGDND------DVDVPIIRYRYRF-
E9AFD0|LmUBA1b      1126 SATVSIDDGDDTVDEVDVETATVCLEIQQ-
```

Figure S2. Amino acid alignments of hUBA1 and the trypanosomatid UBA1s used or mentioned in this study.

Alignments were made with ClustalOmega and coloured with Boxshade. Red residues are identical, blue residues at least 50% similar between sequences. Residues that interact with TAK-243 in ScUBA1 based on PDB 5L6J are highlighted (see Figure 4). Colouring is as follows: residues that do not differ between hUBA1 (and ScUBA1) and the UBA1s of T. brucei are in grey; the gatekeeper residue is in yellow; other residues that were studied and mutated in this study (Figure 5) are in blue; residues that were not mutated but were studied in the structural models (Figure 6) are in green. The lines above the sequences indicate the separate domains with colours as in Figure 1 and Figure S1: the IAD in orange, the FCCH and SCCH in pink, the AAD in red and the UFD in purple. The catalytic cysteine is indicated with an asterisk and the gatekeeper with an arrow.

Gene Locus	size (AA)	ThiF domains	UniProt ID	E1
Tb927.9.12650	1214	2	Q38DE8	UBA1 (Ub)
Tb927.8.2640	1055	2	Q57XC5	UBA1 (Ub)
Tb927.5.3430	796	1	Q57UC3	UBA2 (SUMO)
Tb927.9.4620	467	1	Q38F81	UBA3 (Nedd8)
Tb927.9.6040	387	1	Q38EW4	UBA5 (Ufm1)
Tb927.10.11180	754	1	Q389M1	ATG7 (ATG8)
Tb827.11.2330	505	1	Q386S6	MOCS3 (Urm1, S)
TB927.2.4020	603	1	Q586W2	APPBP1 (Nedd8)
Tb11.02.5410b	295	1	Q384R4	AOS1 (SUMO)

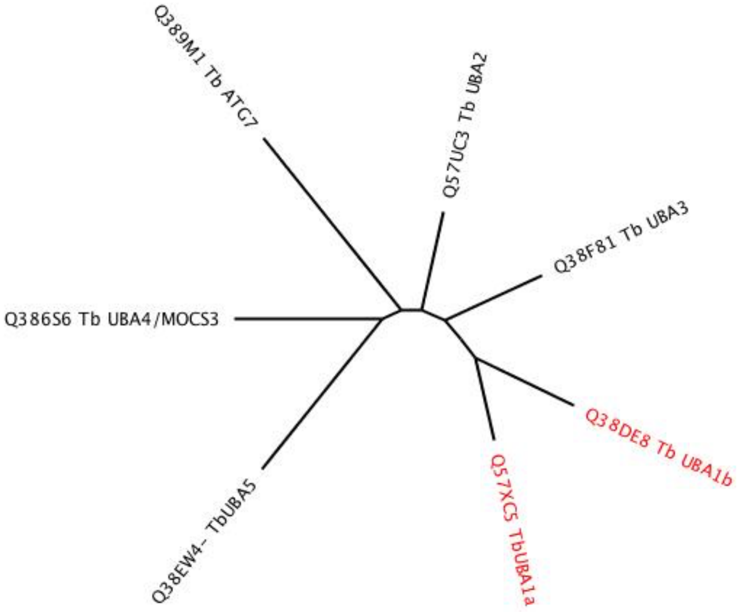

Figure S3. ThiF domain containing proteins in *T. brucei.*

Protein BLAST and ThiF domain (Pfam pf00899) searches in NCBI and TriTrypdp.org identified probable ortholgues of the indicated E1 proteins. AOS1 and UBA2 together form the E1 for SUMO, APPBP1 and UBA3 the E1 for Nedd8. These proteins, excluding AOS1 and APPBP1 without an active adenylation domain (AAD), where aligned in UniprotAlign, phylogenetic analysis was performed with PhyML3.0 (Dereeper A et al. Nucleic Acids Res. 2008 Jul 1;36) and the tree depicted with FigTree software.

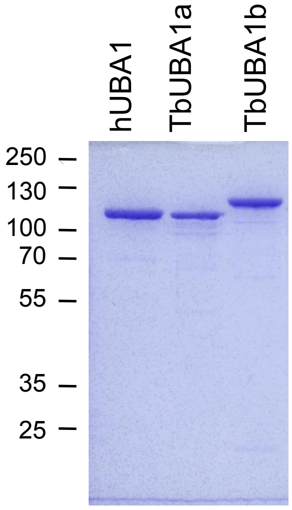

Figure S4. The purified human and T. brucei UBA1 proteins

The indicated UBA1s were purified from E. coli as described in Materials and Methods. A Coomassie stained acrylamide gel is shown.

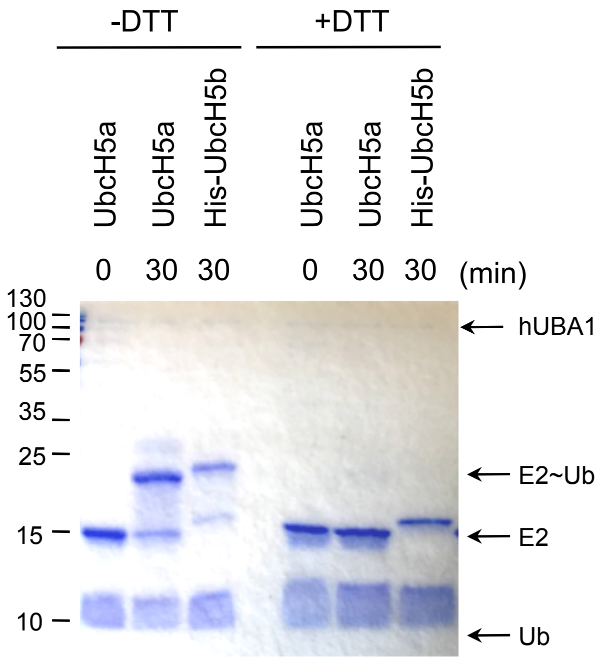

A

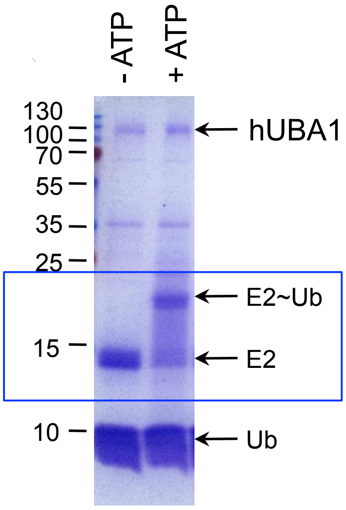

B

Figure S5. The E1-E2 transthioesterifcation reactions used in this study

E1-E2 transthioesterification reactions with hUBA1 were performed as described in Materials and Methods.

A. the transthioesterification reactions were performed with either UbcH5a purified in the laboratory or with commercial His6-UbcH5b (Enzo Life Sciences) for 0 or 30 min as indicated. The reactions were incubated with or without DTT before analysis by SDS-PAGE. The gel was Coomassie stained and dried. The disappearance of the E2~Ub conjugate in the presence of DTT illustrates that ubiquitin was bound to the E2 via a reducible thioester linkage.

B. the transthioesterification reactions were performed in the absence or presence of Mg~ATP (ATP) to show the dependence of the E2~Ub conjugate formation on ATP, as expected. The gel was captured following Coomassie staining using an ImageQuant LAS500 imager as for other gels in this study. The boxed area shows the part of the gels that are shown in Figures 1, 3, 5 and 7 of the main manuscript.

A

```
hu Ub              1  MQIFVKTLTGKTITLEVEPSDTIENVKAKIQDKEGIPPDQQRLIFAGKQLEDGRTLSDYN
T.brucei Ub        1  MQIFVKTLTGKTIALEVEASDTIENVKAKIQDKEGIPPDQQRLIFAGKQLEEGRTLADYN

hu Ub              61 IQKESTLHLVLRLRGG 76
T.brucei Ub        61 IQKESTLHLVLRLRGG 76
```

B

```
UbcH5a                1   MALKRIQKELSDLQRDPPAHCSAGPVGD-DLFHWQATIMGPPDSAYQGGVFFLTVHFPTD
Q57W45 T. brucei E2   1   MALRRIQKELKDLERDPPANTSGGPVNESDLFNWKATIIGPEDSPYAGGLFFLNIHFPSD

UbcH5a                60  YPFKPPKIAFTTKIYHPNINSNGSICLDILRSQWSPALTVSKVLLSICSLLCDPNPDDPL
Q57W45 T. brucei E2   61  YPFKPPKLQFTTKIYHPNINNNGGICLDILKDQWSPALTISKVLLSVCSLLTDPNPDDPL

UbcH5a                120 VPDIAQIYKSDKEKYNRHAREWTQKYAM 147
Q57W45 T. brucei E2   121 VPDIARQYKTDRNAFNKTAMEWTRQYAM 148
```

Figure S6. Alignments of human and T. brucei ubiquitin and E2.

A. Human and T. brucei ubiquitin (Q383T7) differ at 4 residues. Alignments were made with ClustalOmega and coloured with Expasy Boxshade. Identical residues are in red, similar residues in blue. Similarity groups are (G,A), (I,L,M,V), (D,E), (H,K,R) and (S,T).

B. T. brucei expresses an orthologue of the human E2 UbcH5a (E2D1, NP_003329.1) that is 73% identical (Q57W45). Alignment and colouring as in A.

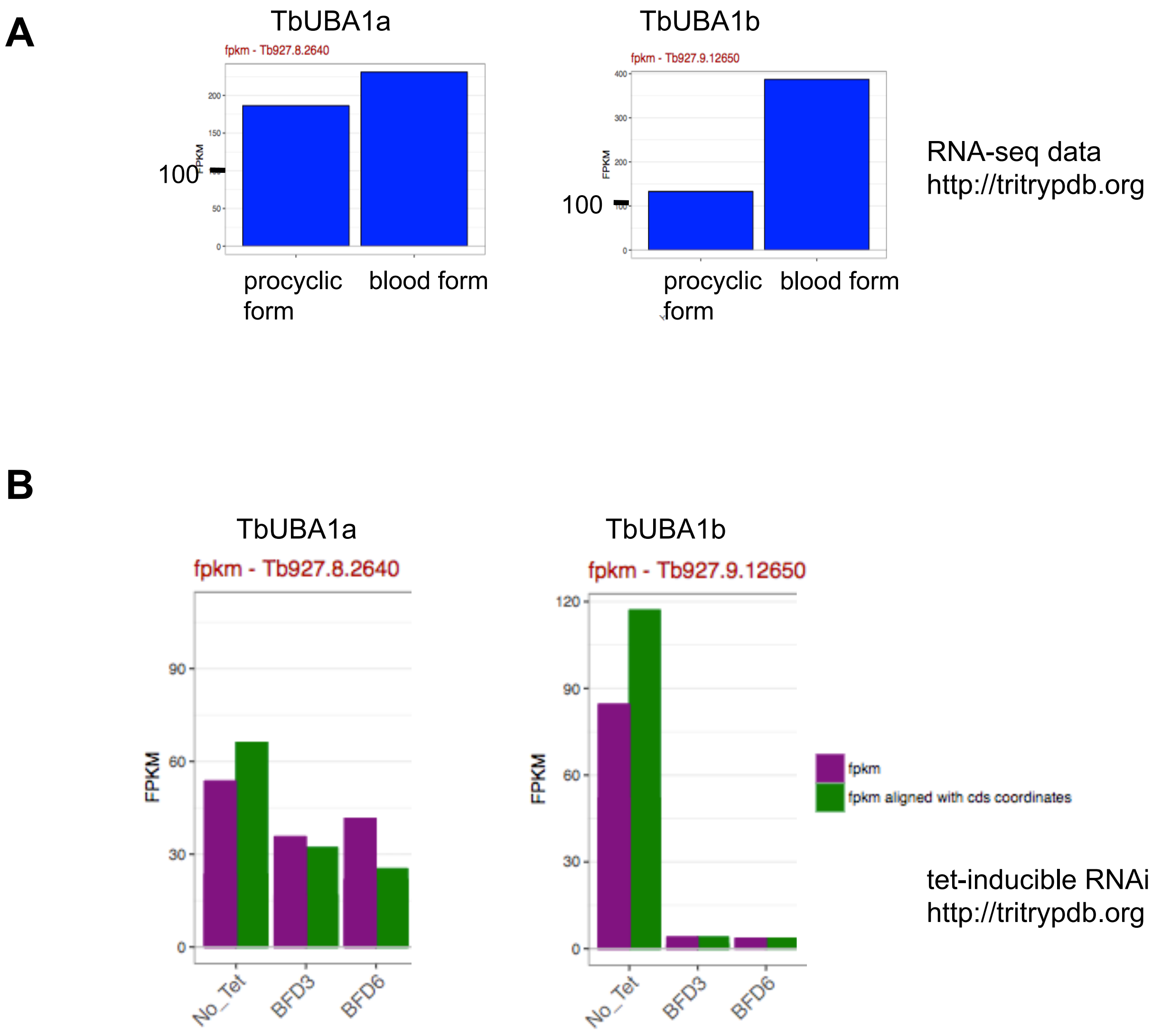

Figure S7. Expression of TbUBA1a and TbUBA1b; Effects of tet-inducible RNAi knockdown of TbUBA1a and TbUBA1b.

A. RNA-seq data from http://tritrypdb.org website showing that TbUBA1a and TbUBA1b are both expressed in the blood form as well as the procyclic form of T. brucei (Siegel T. N. et al. Nucleic Acid Res. 38 (15): 4946 (2010)).

B. Data from http://tritrypdb.org of a tetracycline-inducible RNAi knockdown study of 9891 genes in T. brucei (Alsford S. et al Genome Res 21(6):915 (2011)). Transcript levels before induction of RNAi (No Tet), after 3 days (BFD3) and 6 days (BFD6) are shown.

A

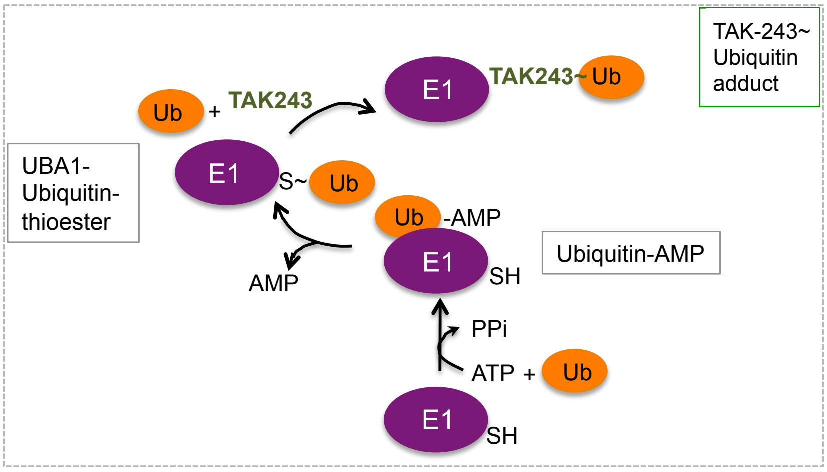

B

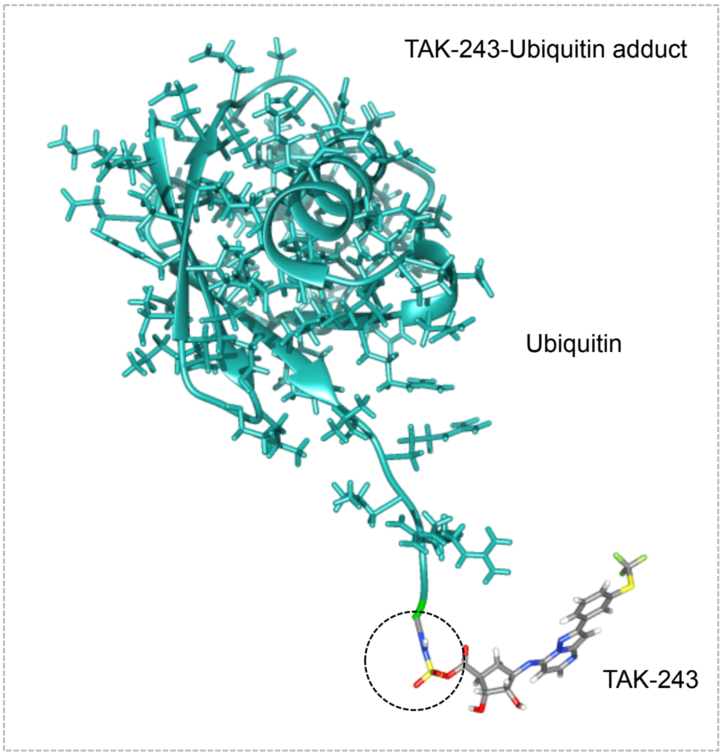

Figure S8. TAK-243 forms a covalent TAK-243~ubiquitin adduct that can not be released from UBA1.

A. In the presence of TAK-243, the thioester-bound ubiquitin on UBA1 is attacked so that a TAK-243~ubiquitin adduct is formed. This substrate-assisted inhibition was elucidated for MLN4924 and UBA3, the active subunit of Nedd8 E1 (Brownell et al. Molecular Cell 37:102, 2010).

B. The TAK-243~ubiquitin adduct bound to ScUBA1 (chain not shown) was taken from PDB 5L6J (Misra et al. Structure 25(7):1120, 2017). The circle indicates the covalent attachment of ubiquitin to the sulfamate nitrogen of TAK-243. The image was generated with UCSF Chimera (Pettersen et al. J Comput Chem 25: 1605-1612, 2004).

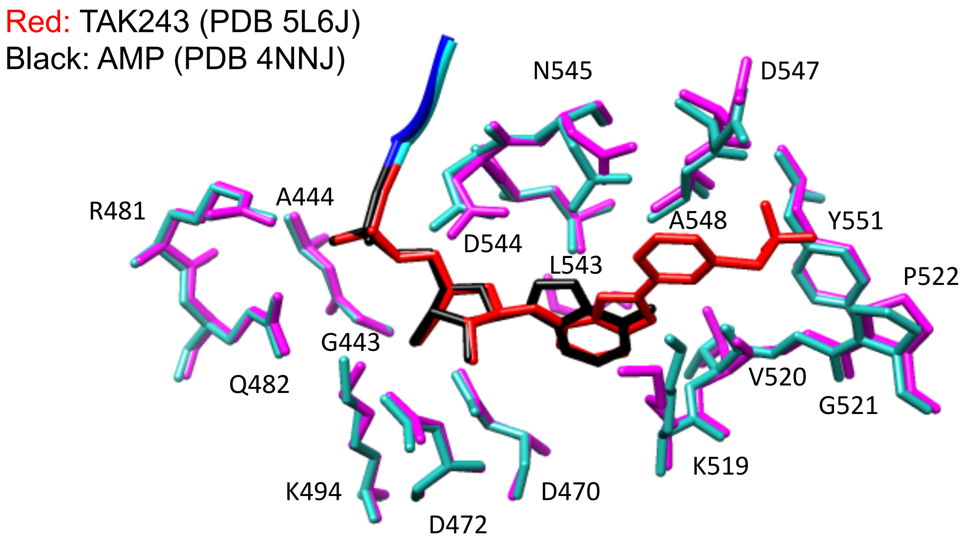

Figure S9. TAK-243 protrudes further into the adenylation site of UBA1 than AMP.

An overlay of residues from ScUBA1/TAK-243~ubiquitin (cyan, PDB 5L6J) with those from ScUBA1/AMP~ubiquitin (magenta, PDB 4NNJ). Only the residues that that interact with TAK-243 in the adenylation site are shown. AMP is in black, TAK-243 in red. Ubiquitin residues are in blue. The image was generated with UCSF Chimera (Pettersen et al. J Comput Chem 25: 1605-1612, 2004).

CLONING PRIMERS

TbUBA1a
For gateway cloning and to introduce an upstream HRV 3C protease cleavage site. This was done in two rounds of PCRs. The first round was with primers Tb26Fw1 and Tb26Rev; the second round with primers GwB1-3C using the product of the first PCR as input (nucleotides for the HRV 3C cleavage site are in italics, nucleotides complementary to TbUBA1a are underlined, recombination sites are in normal text):

Tb26Fw1:	*CTGGAAGTGCTGTTTCAGGGCCCG*ACTAGTGAAGAGCAAAGACGG
Tb26Rev :	GGGGACCACTTTGTACAAGAAAGCTGGGTGTTAAAAATCCTTGTACCTGTA ATAAATTAGAGG
GwB1-3C	GGGGACAAGTTTGTACAAAAAAGCAGGCTTCCTGGAAGTGCTGTTTCAGGG CCCG

TbUBA1b
For restriction enzyme cloning into EcoRI and NotI site of pGEX4T3 (nucleotides complementary to TbUBA1b are underlined):

Tb09EcoFw:	TCCCCGAATTCCCCAAAACATCAACGGGATCGCTTTTCCAATTGTCC
Tb09NotRev:	CCGCGGCCGCTTATTGTTGGATGCGCAAAAGTATTGTAGCGGTTTCAAC

LmUBA1a
For restriction enzyme cloning into EcoRI and NotI site of pGEX4T3 (nucleotides complementary to LmUBA1a are underlined):

LmEcoFw:	TCCCCGAATTCCTTGTCCGAGGAGGAGCAAAAGAGG
LmNotRev:	CCGCGGCCGCTTATTAGAAGCGATAACGGTAGCGGATGATGGGCAC

MUTAGENESIS PRIMERS

TbUBA1a

Q534P-Fw:	GAGAAGGTGGCGCCAGAAACAGAAAATGTG
Q534P-Rev:	CACATTTTCTGTTTCTGGCGCCACCTTCTC
E559D-Fw:	CGCTTGACAATGTAGACAGCCGGAAATATGTTG
E559F-Rev:	CAACATATTTCCGGCTGTCTACATTGTCAAGCG
S560A-Fw:	CGCTTGACAATGTAGAAGCCCGGAAATATGTTG
S560A-Rev:	CAACATATTTCCGGGCTTCTACATTGTCAAGCG
E559D,S560A-Fw:	CGCTTGACAATGTAGACGCCCGGAAATATGTTG
E559D,S560A-Rev:	CAACATATTTCCGGGCGTCTACATTGTCAAGCG

TbUBA1b

S631P-Fw:	GATTACGTGGGACCCAACACGGAACAT
S631P-Rev:	ATGTTCCGTGTTGGGTCCCACGTAATC
E656D,T657A-Fw:	CTTGACAACATGGACGCTCGACTCTATGTTG
E656D,T657A-Rev:	CAACATAGAGTCGAGCGTCCATGTTGTCAAG

Figure S10. Cloning an mutagenesis primers used in this study.

Full length gels for Figures 1 and 3

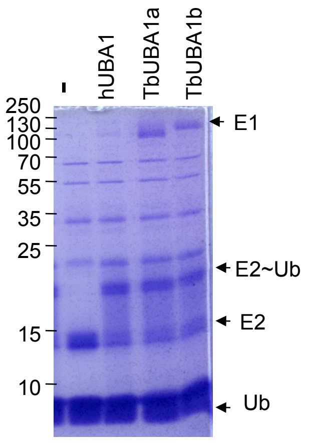

Figure 1D

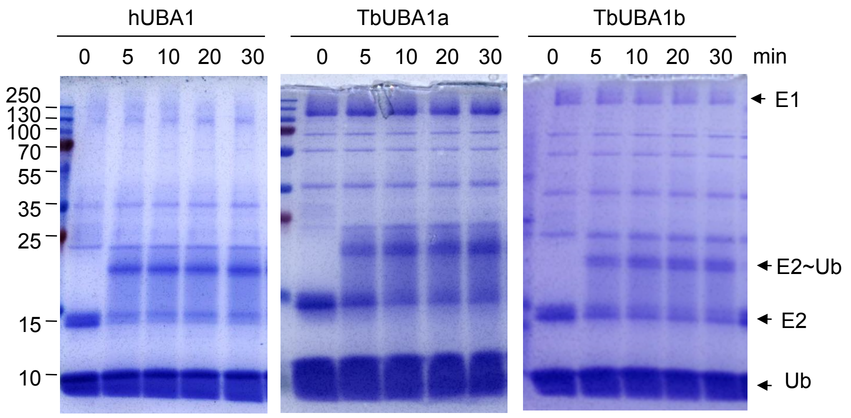

Figure 1E

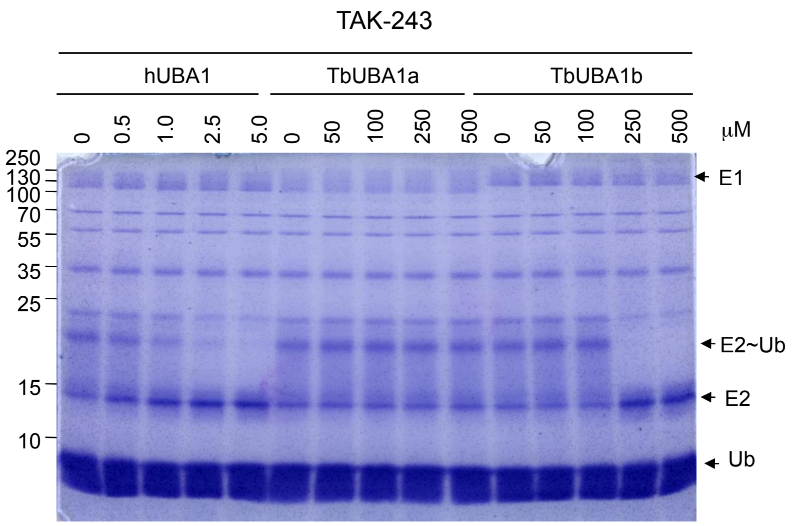

Figure 3A

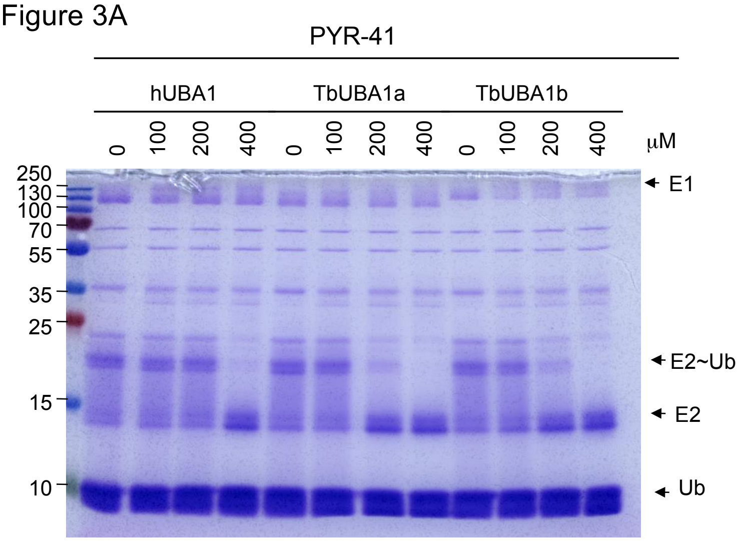

Figure 3C

Full length gels for Figure 5A*

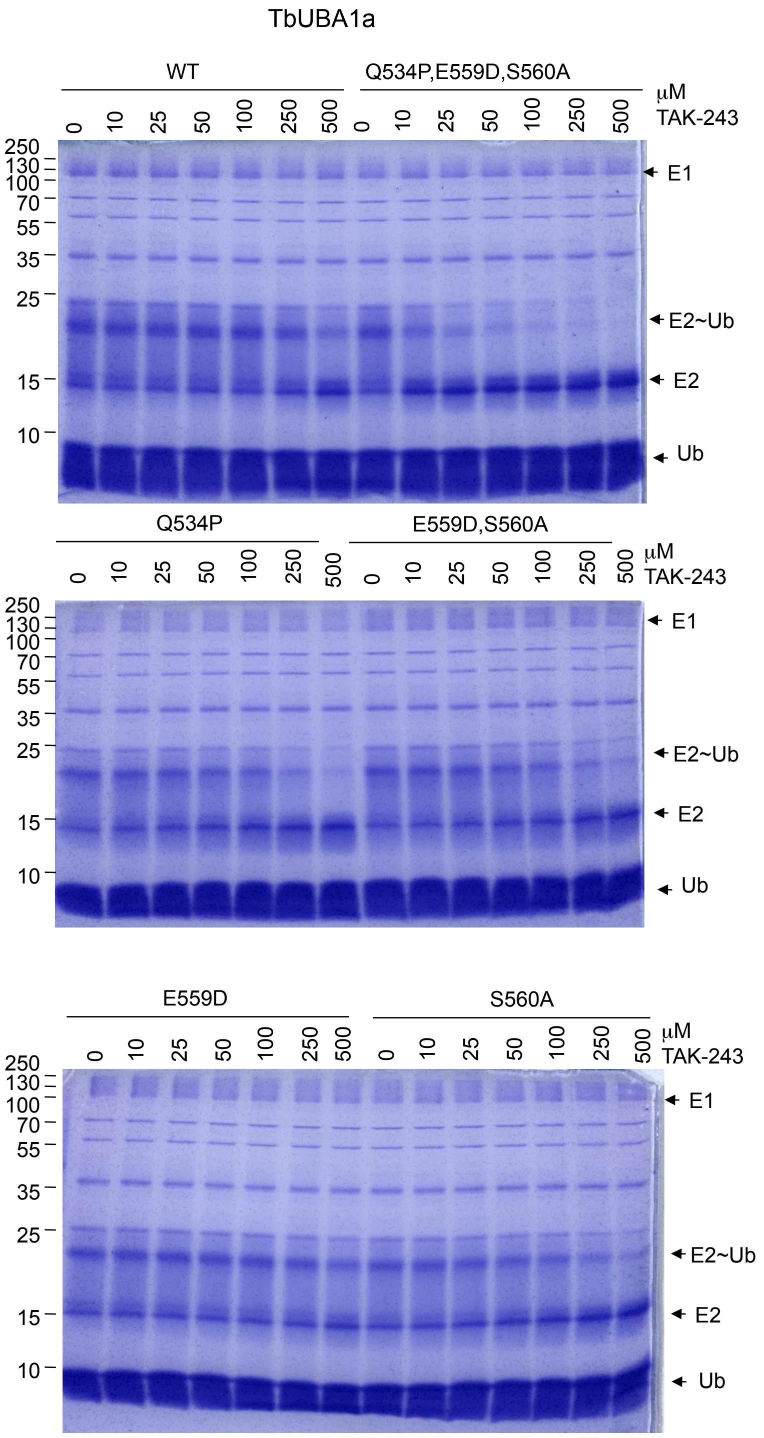

* Note that the order of the mutants on these gels is different from the order in which they are displayed in Figure 5A.

Full length gels for Figure 5B* and C

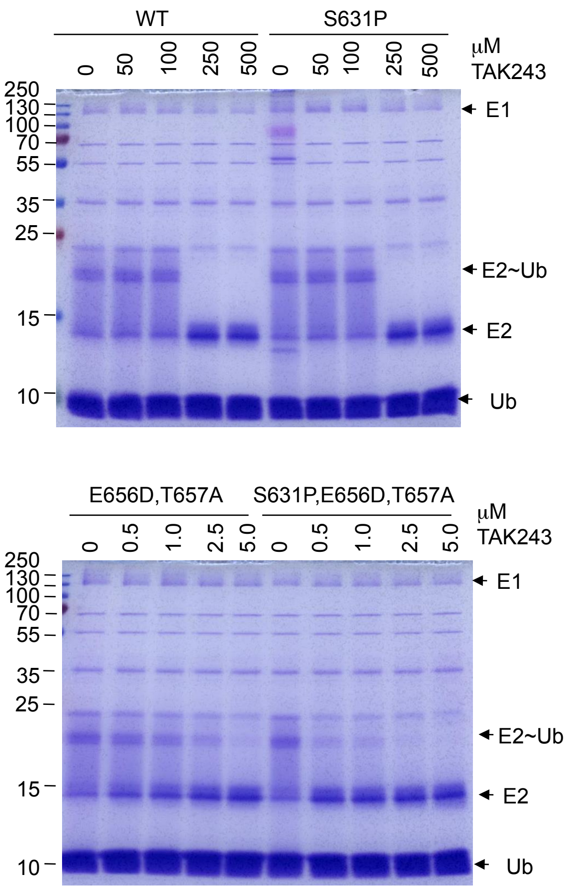

Figure 5B
* Note that the order of the mutants on these gels is different from the order in which they are displayed in Figure 5A.

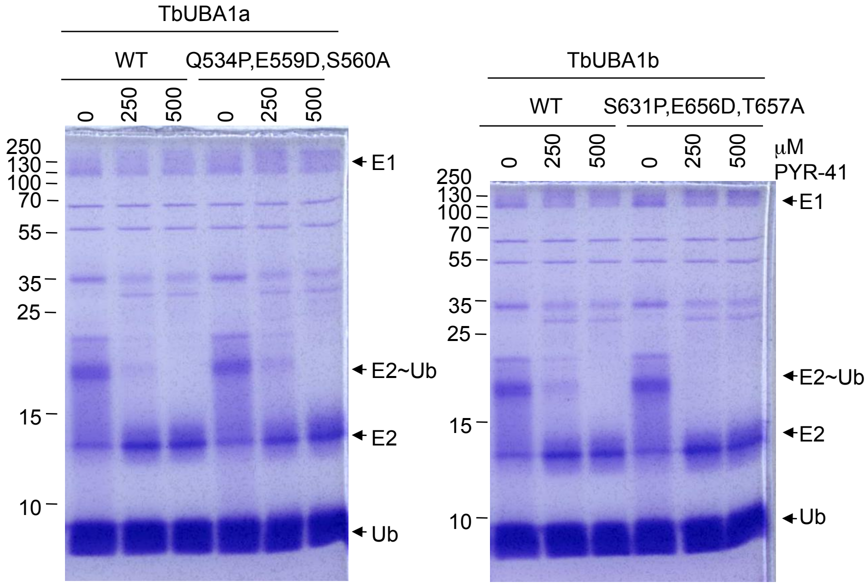

Figure 5C

Full length gel for Figure 7

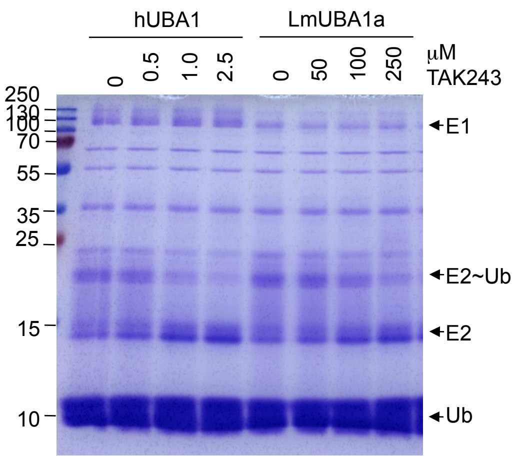

Supplement: Supplementary file 1 — Supplementary Information [file 41598_2019_52618_MOESM1_ESM.pdf]
